# Practical Protocol for Comprehensively Evaluating Sulfur-Fumigation of Baizhi Based on Metabolomics, Pharmacology, and Cytotoxicity

**DOI:** 10.3389/fphar.2021.799504

**Published:** 2022-01-25

**Authors:** Ai-Ping Deng, Chuan-Zhi Kang, Li-Ping Kang, Chao-Geng Lyu, Wen-Jin Zhang, Sheng Wang, Hong-Yang Wang, Tie-Gui Nan, Li Zhou, Lu-Qi Huang, Zhi-Lai Zhan, Lan-Ping Guo

**Affiliations:** State Key Laboratory Breeding Base of Dao-di Herbs, National Resource Center for Chinese Materia Medica, China Academy of Chinese Medical Sciences, Beijing, China

**Keywords:** *Angelica dahurica*, sulfur-fumigation, marker, inflammatory, cytotoxicity, furocoumarin

## Abstract

Sulfur Angelicae Dahuricae Radix (Baizhi) is a common medicinal herb in Asian countries. A practical protocol combining metabolomics, pharmacology, and cytotoxicity was developed to comprehensively evaluate the influence of sulfur-fumigation on the quality of Baizhi. Furocoumarins could be transformed into sulfur-containing compounds during the sulfuring process, among which **1** and **3** were purified with relatively high abundance and identified as 3,4-dihydrobyakangelicin-4-sulfonic acid and (4*R*,12*S*)-3,4-dihydrooxypeucedanin hydrate-4-sulfonic acid (OXH-S), respectively. OXH-S was found to be an addition product of sulfite and oxypeucedanin hydrate (OXH-N). Then, the cytotoxicity and anti-inflammatory activity of OXH-N, OXH-S, and water extracts of sulfured (extraction-S), and unsulfured Baizhi (extraction-N) were evaluated. OXH-S and extraction-S were less toxic than OXH-N and extraction-N, respectively. A comparison of OXH-N with OXH-S and extraction-N with extraction-S showed no significant differences in anti-inflammatory activity. These results suggest that sulfur fumigation can reduce toxicity and does not influence the anti-inflammatory activity of Baizhi, even after chemical composition changes. The proposed protocol based on marker screening, pharmacology, and safety evaluation provides a scientific basis for the standardization and regulation of sulfured Baizhi and other medical materials.

## 1 Introduction

In traditional Chinese medicine (TCM), sulfur fumigation is an extensively used processing method that has been employed for over 100 years. The method allows good moisture-retention, while inhibiting insect and mold growth, as well as preserving the color and freshness of the medicinal products (Wu et al., 2012; Duan et al., 2016). Sulfur fumigation involves burning sulfur in a closed space, wherein fresh medicinal materials are stocked, during which sulfur dioxide forms sulfite upon reacting with water molecules present in fresh medicinal materials. The strong reducing power of sulfite can specifically exterminate eggs, larvae, pupae, and adult moths, thereby preventing insect infestation. Additionally, sulfite is also known to inhibit the activity of oxidases in food, consequently removing dark discoloration and preventing browning, thereby helping to maintain the visual appeal of foods (Zhan et al., 2018). However, despite the beneficial effects of sulfur fumigation, several researchers have expressed concerns regarding its negative impact on the safety and effectiveness of TCM, considering the presence of residual sulfur dioxide and the chemical changes it may introduce (Wu et al., 2012; Kong et al., 2017; Lou et al., 2017; Sun et al., 2017). Nevertheless, due to insufficient evidence to prove that sulfur fumigation can degrade the quality of TCMs, these concerns remain debatable.

Sulfur fumigated TCM contains many sulfur-containing compounds (Deng et al., 2019). The present study focused on the pharmacology and toxicology of sulfured TCM extraction (Cheng et al., 2010; Jiang et al., 2011; Ma et al., 2017). However, the differences in the pharmacology and toxicology of these compounds, and of their related substrates, remain unexplored. Owing to the large number of isomers of sulfur-containing compounds and their high acidity and polarity, their efficient separation and purification remains a challenge. To date, only three sulfur-containing compounds have been purified and identified in sulfured TCMs, namely paeoniflorin sulfonate in sulfured Paeoniae Radix Alba (Kong et al., 2017), (3β, 6α, 12β)-3, 12-dihydroxydammar-25-ene-6, 20-diylbis-β-D-glucopyranoside, 24-sulfonic acid in sulfured Ginseng Radix et Rhizoma (Zhang et al., 2018), and *p*-hydroxybenzyl hydrogen sulfite in sulfured Gastrodia Rhizoma (Kang et al., 2017). Studies elucidating the efficacy and toxicity of these sulfur-containing compounds are crucial to obtain a more comprehensive understanding of their effects on TCM.

Among the sulfured TCMs, Angelica Dahuricae Radix (Baizhi) is the most commonly available TCM on the market. Baizhi, the dried root of *Angelica dahurica* (Hoffm.) Benth. & Hook. f. ex Franch. & Sav. (Apiaceae), is an important medicinal plant that has been widely cultivated across several Asian countries, particularly China, for thousands of years (Zhao et al., 2016). It is commonly used to treat superficial pathological conditions, such as common could and cold, pain relief, leukorrhea, clearing the nasal cavity, relieving swelling, and evacuating pus; thus, Baizhi has been prescribed to treat headaches, toothaches, abscesses, nose congestion, acne, and furunculosis (Pharmacopoeia of People’s Republic of China, 2020). All these outcomes are related to the anti-inflammatory activity (Yang et al., 2015; Yang et al., 2020) of Baizhi, which is mainly conferred by the constituent coumarins, and specifically the furanocoumarins (Chen et al., 2015). However, Baizhi also exerts other pharmacological effects, such as anti-microbial (Yang et al., 2020) and anti-cancer (Luo et al., 2011; Zheng et al., 2016) activities. Sulfur fumigation is frequently used in Baizhi processing, which imparts some distinct changes in the chemical properties of the final product, including a drastic reduction in oxypeucedanin; a significant decline in imperatorin, cnidilin, and isoimperatorin concentration; and emergence of various new compounds (Fan et al., 2012; Lu, 2013). However, these new chemical compounds produced in sulfured Baizhi have not been evaluated, especially regarding their structure, safety, and efficacy. Therefore, in this study, we developed a protocol that combines metabolomics, pharmacology, and cytotoxicity profiling to evaluate whether sulfur fumigation of Baizhi is beneficial or harmful in terms of the overall quality of the medicinal product. The proposed protocol provides a possibility to systematically clarify the effect of sulfur fumigation on the chemical components, effectiveness, and safety of TCM.

## 2 Materials and Methods

### 2.1 Materials and Instruments

#### 2.1.1 Crude Baizhi Materials

Three fresh Baizhi samples (unsulfured Baizhi 1–3) were collected from Bozhou, Anhui Province, China (33°57′32.41″N, 115°49′57.89″E) on August 13, 2018. For ultra-performance LC quadrupole time-of-flight MS (UPLC-Q-TOF-MS) analysis, sulfured Baizhi 1–3 samples were prepared as reported previously to obtain the material with sulfur dioxide residue at approximately 750 mg/kg (Kang et al., 2017). The fresh Baizhi samples were washed and cut into 2-mm slices. Then, they were divided into two groups (unsulfured group and sulfured group); each group of samples was placed in two plastic boxes (60 × 40 × 30 cm) with six holes (*d* = 0.5 cm) on the surface. Next, 20 g of sulfur was then lighted with 12 ml alcohol in the box for sulfured groups. 12 ml alcohol was lighted in the box of unsulfured group. Twenty-4 hours later, all samples were dried at 40°C, and ground to a fine powder (50 mesh) using a pulverizer (Tianjin Taisite Instrument Co., Tianjin, China), and were stored at 4°C for further analysis. Sulfured Baizhi decoction pieces (10 kg) for the purification of markers of sulfured Baizhi were purchased at the Bozhou TCM trading market (Bozhou, Anhui Province, China) on October 23, 2019. All samples were identified as *A. dahurica* (Fisch. ex Hoffm.) Benth. et Hook. f. by Professor Lan-Ping Guo.

#### 2.1.2 Chemicals

Analytical-grade methyl red, acetate, alizarin red S, and sodium sulfate were obtained from Shanghai Macklin Biochemical Co., Ltd. (Shanghai, China). Analytical-grade hydrogen peroxide (30%) was obtained from Beijing Chemical Works (Beijing, China) and 0.01 mol/L sodium hydroxide solution for volumetric analysis was obtained from the National Institute of Metrology (Beijing, China). Deionized water (18.2 MΩ cm) was prepared using a Milli-Q system (Millipore, Billerica, MA, USA). Liquid chromatography (LC)-grade acetonitrile (Merck KgaA Co., Darmstadt, Germany), LC/mass spectrometry (MS)-grade formic acid (Thermo Fisher Scientific, Waltham, MA, USA), analytical-grade methanol (Sinopharm Chemical Reagent Co., Ltd., Shanghai, China), and lipopolysaccharide (LPS; 017M4112V, Sigma, St. Louis, MO, USA) were also used. Oxypeucedanin hydrate (OXH-N) with high purity (95%) was purchased from Shanghai Yuanye Biotechnology Co., Ltd. (Shanghai, China).

#### 2.1.3 Instrumentation

A BSA224S analytical balance (Sartorius, Gottingen, Germany), centrifuge 5415D (Eppendorf, Hamburg, Germany), SB-800DTD ultrasonic processor (Ningbo Scientz Biotechnology, Ningbo, China), and 0.22 µm polytetrafluoroethylene filter (Pall Corporation, Farmingdale, NY, USA) were used for UPLC-Q-TOF-MS analysis. An EYELA N-3010 rotary evaporator (Tokyo Rikakikai, Ltd. Tokyo, Japan) and SP825L macroporous resin (Mitsubishi Chemical, Tokyo, Japan) were used to purify compound **1** and compound **3**. A dissecting microscope (SZX7, Olympus, Tokyo, Japan), charge-coupled device camera (VertA1, Shanghai Tusen Vision Technology Co., Ltd., Shanghai, China), MULTIZOOM AZ100 microscope (Nikon, Tokyo, Japan), microinjector (PCO-1500, Zgenebio, Taipei, Taiwan), micropipette puller (PC-10, Narishige, Tokyo, Japan), 6-well plate (NEST Biotech Co., Ltd., Wuxi, China), and methylcellulose (079K0054V, Sigma) were used to evaluate anti-inflammatory activity and cytotoxicity.

### 2.2 Screening of Markers to Differentiate Sulfured and Unsulfured Baizhi

#### 2.2.1 Sample Preparation

The powdered sulfured and unsulfured samples were ultrasonically extracted with 70% ethanol for 90 min at 25°C and 40 kHz using an ultrasonic processor to prepare samples with a concentration of 200 mg/ml. After centrifugation at 13,800 × *g* at 25°C for 10 min, each sample extract was filtered through a 0.22 µm membrane filter prior to UPLC-Q-TOF-MS analyses, and 50 µL of each extract was mixed to prepare a quality control sample.

#### 2.2.2 UPLC Conditions

UPLC separation was performed on an Acquity UPLC I-Class system (Waters Co., Milford, MA, USA). A Waters UPLC Acquity HSS T3 column (2.1 × 100 mm, 1.8 µm) was used for chromatographic separation. The mobile phase consisted of (A) 0.1% (v/v) formic acid in deionized water and (B) acetonitrile containing 0.1% (v/v) formic acid. The linear elution gradient was optimized as follows: 5% B (0–2 min), 5→30% B (2–7 min), 30→45% B (7–12 min), 45→60% B (12–25 min), 60→90% B (25–30 min), 90→98% B (30–30.05 min), and 98% B (30.05–33 min). The parameters were set as follows: flow rate, 0.5 ml/min; injection volume, 1 µL; column temperature, 40°C; UV, 200–400 nm.

#### 2.2.3 MS Conditions

Chemical profile analysis was performed on a Waters Xevo G2-S Q-TOF-MS system (coupled with electron spray ionization) in both negative and positive electrospray ionization modes. The Q-TOF-MS parameters were set as follows: acquisition mass range, 50–1,500 Da; scan time, 0.5 s; capillary voltage, 2.0 kV; cone voltage, 40 V; source temperature, 100°C; desolvation gas temperature, 280°C; cone gas flow rate, 50 L/h; and desolvation flow rate, 650 L/h. The collision energy was set to 6 eV (trap) for low-energy scans and 30–60 eV for high-energy scans, and all MS data were acquired using leucine-enkephalin at a concentration of 200 ng/ml to ensure accuracy and reproducibility. The accurate mass weights were 554.2615 and 556.2771 in negative and positive ion modes, respectively.

### 2.3 UPLC-QTOF-MS-Guided Marker Isolation and Purification

UPLC-QTOF-MS-guided isolation has been described previously for sulfur-containing derivatives (Zhang et al., 2018). Sulfured Baizhi (10 kg) was prepared and extracted for 30 min at 25°C using an SB-800DTD ultrasonic processor (with an online filtration system) with 80% ethanol aqueous solution (70 L × 3 times). After filtration through a double gauze layer, the extracted solution was concentrated using an EYELA N-3010 rotary evaporator at 50°C. The condensate was then suspended in water (5 L) and partitioned with petroleum ether and ethyl acetate (5 L × 3 times).

The water fraction was subjected to SP825L macroporous resin column chromatography and eluted with aqueous ethanol (0, 15, 30, 50, and 100%; a three-column volume (15 L) of each solvent was used). The purification procedures were guided by UPLC-Q-TOF-MS. Then, 30% ethanol was purified by 1,260 Infinity HPLC (Agilent Technologies Inc., CITY, CA, USA). Finally, 2 mg of compound **1** and 324 mg of compound **3** were obtained. The purity of compounds **1** and **3** were found to be more than 80 and 95%, respectively. The purity was determined by UPLC.

### 2.4. Structural Analysis of Compounds 1 and 3

Compounds **1** and **3** were prepared at a concentration of 100 μg/ml in 70% methyl. Next, 1 µL of each compound was injected for UPLC-Q-TOF-MS to obtain high-resolution MS data. Compounds **1** (2 mg) and **3** (5 mg) were dissolved in 1 ml D_2_O and transferred to a nuclear tube (*d* = 2 mm) for ^1^H nuclear magnetic resonance (NMR), ^13^C NMR, heteronuclear single quantum coherence (HSQC), and heteronuclear multiple bond correlation (HMBC), the spectra of which were recorded on a Bruker Avance III 600MH spectrometer (Billerica, MA, USA) operating at 600 MHz for ^1^H and 150 MHz for ^13^C, with tetramethylsilane used as an internal standard.

Mosher’s method (Wang et al., 2001) was used to determine the stereoscopic configuration of C-11. To prepare (*R*)- and (*S*)-(**3**)-α-methyl-α-trifluoromethylphenyl lactic acid (MTPA) esters of compound **3**, a solution of compound **3** (4 mg) in pyridine-d5 (0.5 ml) and (*R*)-(-)-MTPA Cl (4 µL) was left to stand overnight at 20–30°C with stirring under argon protection to obtain (*R*)-(**3**)-MTPA ester. Similarly, the (*S*)-(**3**)-MTPA ester was obtained from compound **3** using (*S*)-(-)-MTPA Cl.

For electronic circular dichroism calculations, a conformation search of compound 3 was performed in Molclus (version 1.9.9.4) based on the xtb program using the GFN-xTB method (Lu, 2020; Lu, 2021). Conformers with a relative energy less than 3 kcal/mol were selected for optimization at the B3LYP/6-31g(d) level in the gas phase using the Gaussian 09 program (Frisch et al., 2013). Six conformers were then selected for (4*R*,12*S*)-**3** and five conformers were selected for (4*S*,12*S*)-**3** based on their Boltzmann distribution (>1%). The theoretical calculation of electronic circular dichroism was conducted in methanol using the time-dependent density functional theory at the CAM-B3LYP/6-31 + g (d, p) level for the optimized conformers of **3**. The rotational strengths for 50 excited states were calculated.

### 2.5 Cytotoxicity Evaluation

#### 2.5.1 Preparation of Experimental Samples

Sulfured and unsulfured Baizhi (20.02 g) were extracted for 1 h by soaking in water (20× amount of unsulfured and sulfured Baizhi) for 1 h, followed by centrifugation at 13,800 ×*g* at 25°C for 10 min. The supernatant was concentrated and freeze-dried to obtain 3.0 g extractions of unsulfured (extraction-N) and 3.1 g of sulfured (extraction-S) Baizhi. The drug extract ratios were 0.15 g/g. OXH-N is oxypeucedanin hydrate. OXH-S was obtained as described in *UPLC-QTOF-MS-Guided Marker Isolation and Purification*.

OXH-N, OXH-S, extraction-N, and extraction-S (40 mg) were dissolved in dimethyl sulfoxide (biological grade; Beyotime Biotechnology Co., Ltd., Shanghai, China). RPMI-1640 culture medium (Gibco, Grand Island, NY, USA) was added to the target concentration (described in Cell Viability *Evaluated Using MTT Assays and Determination of MTC*). This preparation was filtered through a sterile filter membrane and stored at 4°C.

#### 2.5.2. Cell Viability Evaluated Using MTT Assays

PC12, L02, and HK2 cells, which were kindly provided by the Stem Cell Bank of the Chinese Academy of Sciences, Shanghai, China, were cultured as previously reported (Huang et al., 2020). All cells were maintained in RPMI 1640 medium supplemented with 10% (v/v) FBS and, 100 U/mL penicillin–100 μg/ml streptomycin (Product No: C0222, Beyotime Biotechnology, Shanghai, China), at 37°C with 5% CO_2_ in a humidified incubator. Each of the cell strains were divided into 13 groups: one of the groups served as the control (no treatment with drugs), whereas the other 12 groups were treated with three different concentrations of OXH-N, XOH-S, extraction-N, or extraction-S.

Cell viability was determined using the Cell Counting Kit (CCK)-8 assay (An et al., 2016). Cell suspensions in the logarithmic growth phase were inoculated in 96-well plates (200 µL/well, 3,799 type, Corning Incorporated, NY, USA) at a concentration of 8,000 cells/well. The cells were pre-incubated in the plate in a humidified incubator (37°C, 5% CO_2_) for 12 h. Then different concentrations of drugs (L02 cells: 50, 100, 200 μg/ml; HK2 and PC12 cells: 200, 400, 800 μg/ml) were added. After 24 h, the medium was discarded. CCK-8 solution (10 µL) and culture medium (10 µL) were added to each well of the plate. The plate was incubated for an additional 2 h in an incubator (37°C, 5% CO_2_), after which, the absorbance was measured at 450 nm using a microplate reader (Bio-Rad, Hercules, CA, USA). Cell viability was expressed as a percentage of the control. The growth inhibition rate was calculated as follows:

$$\text{Cell}\;\text{viability}\left(\text{\%}\right)=\frac{\text{absorbance}\;\text{of}\;\text{treatment}\;\text{group}}{\text{absorbance}\;\text{of}\;\text{control}\;\text{group }}\times 100\%$$

Growth inhibition rate(%)=1−Cell viability(%)

To determine the maximum tolerated concentration (MTC) of each drug, cells were treated with 1, 50, 100, 200, 400, 800, and 1,000 mg/L of each drug. At least three independent experiments were performed.

### 2.6 Evaluation of Anti-inflammatory Effect of Extraction-N, Extraction-S, OXH-N, and OXH-S

Extraction-N and extraction-S (prepared in *Preparation of Experimental Samples*) were dissolved in water to prepare the mother liquor at a concentration of 2000 μg/ml. These samples were diluted with water to the target concentrations.

#### 2.6.1 Animal Maintenance

Transgenic Tg (MPX: EGFP) zebrafish (Renshaw et al., 2006) were maintained according to standard protocols (Nusslein-Volhard and Dahm, 2002). Zebrafish were housed in 3.5 L acrylic tanks under the following conditions: test solution, 200 mg of instant sea salt and 50–100 mg of CaCO_3_ dissolved in 1 L of reverse osmosis water; conductivity, 450–550 μS/cm; pH, 6.5–8.5. The license number of the laboratory zebrafish was SYXK (Zhejiang) 2012-0171. All experiments were performed in accordance with the requirements of the International Association for Assessment and Accreditation of Laboratory Animal Care (certification number: 001458). All experiments were approved by the Institutional Animal Care and Use Committee at Hunter Biotechnology, Inc. In total, 810 transgenic Tg (corola: MPX) zebrafish larvae at 3 days post-fertilization (dpf) were used for the experiments, which are described in *Determination of MTC and Establishment of Inflammation Model and Drug Administration*.

#### 2.6.2 Determination of MTC

In total, 360 zebrafish were randomly divided into 12 groups with 30 zebrafish in each group including a blank control group, modal control group, and 10 experiment groups (treated with different drugs at different dosages). Zebrafish larvae (3 dpf) were exposed to different concentrations (125, 250, 500, 1,000, and 2000 μg/ml) of extraction-N and OXH-N for 1 h in the test solution. A microinjector (PCO-1500, Zgenebio) was used to inject LPS into the yolk sac of each zebrafish, except for those in the blank control group. After 2 h, the phenotype and mortality of zebrafish were observed and recorded to determine the MTC.

The proportions of normal, malformed, and dead zebrafish larvae were also recorded at 24 and 48 h using an SZ780 stereo microscope (Optec, Chongqing, China).

#### 2.6.3 Establishment of Inflammation Model and Drug Administration

The inflammation model was established according to standard protocols (Loynes et al., 2010). In total, 450 zebrafish were randomly divided into 15 groups with 30 zebrafish in each group as follows: blank control group (with no special treatment), model control group, positive control group (treated with 17.9 μg/ml indomethacin), and 12 experiment groups (treated with different drugs at different dosages). All zebrafish were incubated in 6-well plates in a 28°C incubator, with a capacity of 3 ml per well. Experiments were performed as described in *Determination of MTC*. The doses of each drug were 55.6, 167, and 500 μg/mL. A microinjector (PCO-1500, Zgenebio) was used to inject LPS into the yolk sac of each zebrafish, except for those in the blank control group. Two hours after administration, 10 zebrafish were randomly chosen from each group and photographed under a continuous zoom fluorescent microscope with electric focusing (AZ100, Nikon). The number of fluorescent neutrophils at the site of inflammation was counted by eye. The anti-inflammatory effects of each drug on LPS-induced inflammation in zebrafish were evaluated by analyzing the number of neutrophils. The inflammatory regression effect (a lower value indicates a stronger anti-inflammatory effect) was calculated based on the number of neutrophils. The formula used for calculation was as follows:

$$\text{Extinction}\;\text{effect}\;\text{of}\;\text{inflammation}\left(\text{\%}\right)=\frac{\text{N}\left(\text{model}\;\text{control}\;\text{group}\right)-\text{N}\left(\text{test}\;\text{group}\right)\;}{\text{N}\left(\text{model}\;\text{control}\;\text{group}\right)\;}\times 100\%$$

#### 2.6.4 Statistical Analysis

The cytotoxicity and anti-inflammatory results are presented as the means ± standard deviation (Huang et al., 2020). One-way analysis of variance with Bonferroni’s multiple comparisons post-hoc test was used to determine the differences between the control and treated groups using GraphPad Prism version 8.0 software (Graph Pad, Inc., San Diego, CA, USA); *p* < 0.05 was considered to indicate statistically significant results.

### 2.7 Ethical Approval

The zebrafish facility and laboratory at Hunter Biotechnology, Inc. are accredited by the AAALAC. After the experiments, all zebrafish were anesthetized and euthanized with 0.25 g/L tricaine methane sulfonate according to the American Veterinary Medical Association requirements for euthanasia using anesthetic. This study was approved by the Institutional Animal Care and Use Committee at Hunter Biotechnology, Inc. with approval number 001458.

### 2.8 Stability of Marker and Sulfur Dioxide Residue During Boiling

Twenty-five microliters of 0.011 mg/ml OXH-S (compound **3**) in water was added to three clean round-bottomed flasks and refluxed and decocted at 100°C for 1 h. Water was added to replace that lost before boiling. The sample solutions were then filtered through a 0.22 µm membrane filter prior to analysis, and 1 µL was injected into the UPLC-PDA system to detect concentrations of OXH-N and OXH-S before and after decoction.

The content of sulfur dioxide residue in the non-boiled and boiled samples was determined using a standard method documented in the Chinese Pharmacopeia 2020 version (Part four) Appendix 2331 (Pharmacopoeia of People’s Republic of China, 2020). The non-boiled group was a sulfured Baizhi powder sample. The boiling group samples were obtained as follows: 10 g of sulfured Baizhi was extracted in 400 ml of water for 1 h. The samples were then filtered using quantitative filter paper. The filtrate was used as the boiling group sample. Three replicates were prepared for each treatment sample and injected into the UPLC to detect OXH-S and OXH-N.

## 3 Results and Discussion

### 3.1 Screening of Markers to Differentiate Sulfured and Unsulfured Baizhi

**FIGURE 1:**
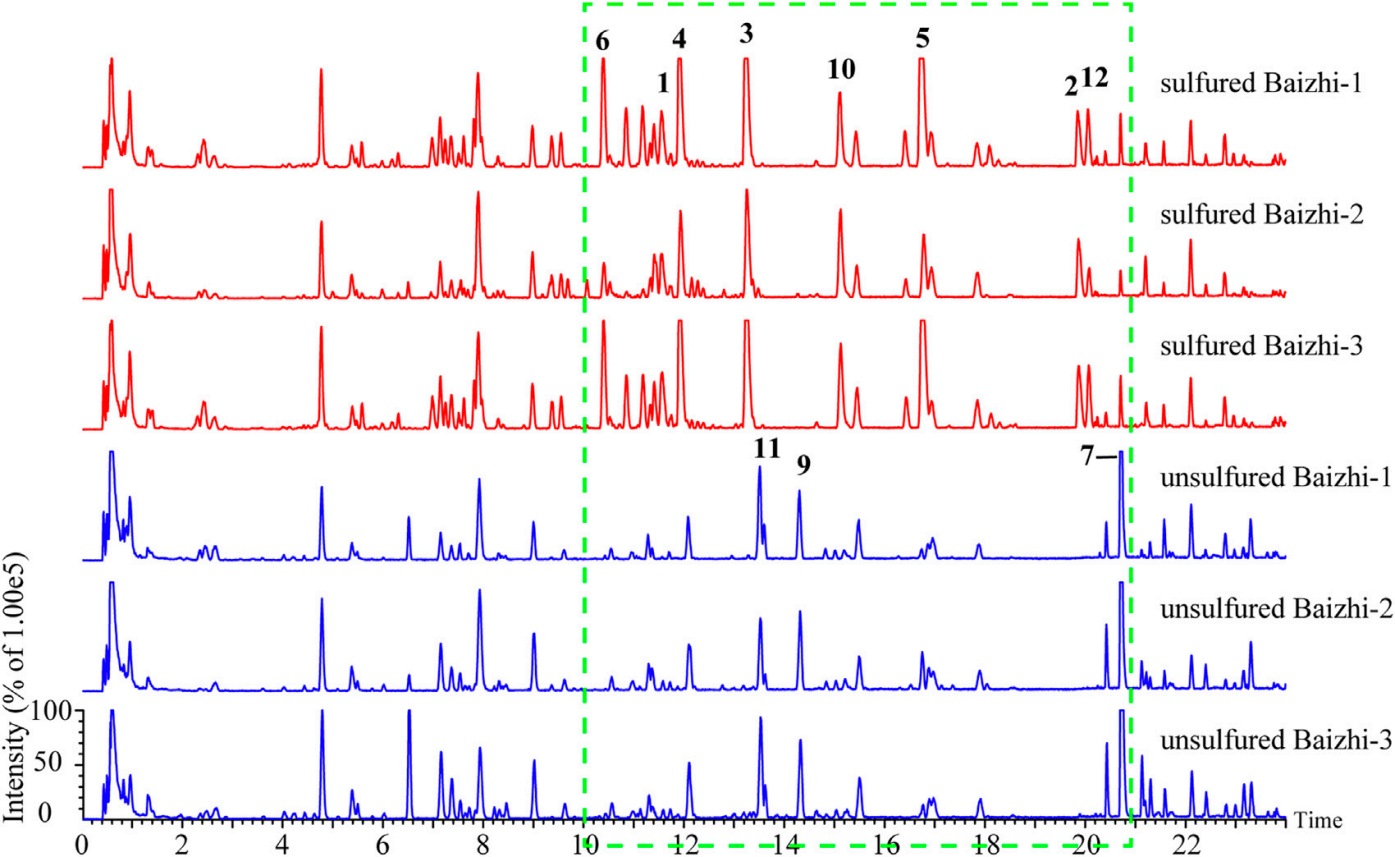
Total ion chromatograms of sulfured and unsulfured Baizhi in negative mode (compounds **1**–**12** are the significantly different compounds).

The chemical constituents of Baizhi were separated within 23 min in both the positive and negative ion modes. Significant differences were observed between unsulfured and sulfured Baizhi in the negative mode. Therefore, the experiment was conducted in the negative mode. Some components of Baizhi were notably affected by sulfur fumigation, such as compounds with retention times between 9 and 21 min (Figure 1).

**FIGURE 2:**
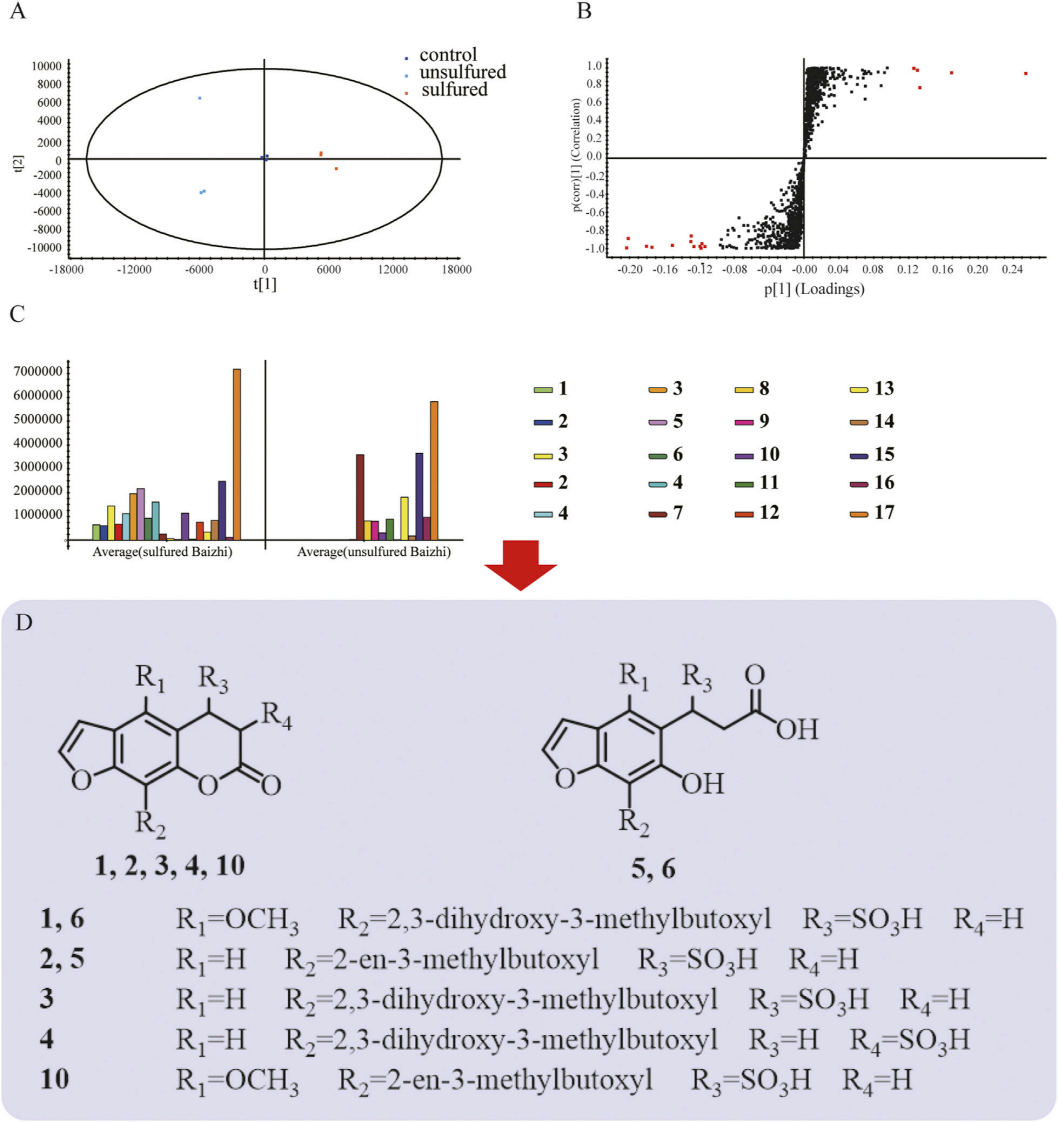
Screening of markers. **(A)** Principal component analysis; **(B)** OPLS-DA results of sulfured and unsulfured Baizhi; **(C)** markers of sulfured and unsulfured Baizhi; **(D)** structures of the markers.

For screening the markers of sulfured and unsulfured Baizhi, raw data obtained using Masslynx V4.1 were imported into QI software (Waters Corp.), and [M-H]^−^, [M-H2O-H]^−^, [M + HCOO]^−^, [2M-H]^−^, [M + Na-2H]^−^, [M + Cl]^−^, [M + K-2H]^−^, and [2M + HCOO]^−^ were selected as addition ions. The samples were categorized into sulfured group, unsulfured group, and quality control group, and all data peaks were aligned with that of the quality control group. A total of 6,002 peaks were obtained, and the compounds with *p* value ≤0.01, minimum coefficient of variation ≥30, and max fold-change ≥2 were selected for principal component analysis. The quality control samples in the PCA score plot suggested good stability and repeatability of LC–MS data. The sulfured and unsulfured samples were well-separated and divided into two groups as shown in Figure 2A. The data were imported into EZinfo 3.0 (Waters Corp.) for orthogonal partial least squares discrimination analysis. In the S-plot diagram (Figure 2B), points marked in red were selected as the differentiation components between the two groups. As shown in Figure 2C, compared with unsulfured samples, the significantly increased compounds contained sulfur, particularly sulfur-containing coumarins, including compounds **1–6**, **10**, and **12**. The compounds decreased significantly were compounds **7**, **9**, and **11**.

The configuration of compound **1** (adduction product of byakangelicin and sulfurous acid) and compound **3** (adduction product of oxypeucedanin hydrate and sulfurous acid) are described in *Structural Analysis of Compounds 1 and 3*. Based on ion fragment pathway of compound **1** and **3**, the structures of other sulfur-containing derivatives were proposed. The ion fragment pathway of compound **1** and **3** are show in Supplementary Figures SA1, 2. Compound **4** had the same molecular and fragment pathway of compound **3**, so **4** was regarded as the other addition product of oxypeucedanin hydrate and sulfurous acid, and was named 3,4-dihydrooxypeucedanin-3 hydrate sulfonic acid. The fragment pathway of compound **4** is shown in Supplementary Figures SA3. Compound **2** had one more H_2_SO_3_ compared with imperatorin, so it was speculated to be 3,4-dihydroimperatorin-4-sulfonic acid; the fragment pathway is shown in Supplementary Figure SA4. Similarly, compound **10** and **12** were identified as dihydrobyakangelicol sulfonic acid and 3,4-dihydrophellopterin-4-sulfonic acid, respectively. The fragments of **10** and **12** are shown in Supplementary Figures SA5, 6. Compound **6** had similar fragmentation as compound **1** (Supplementary Figures SA7), but had one extra H_2_O compared with compound **1**. Considering that lactone bond would not be stable if C3-C4 double bond is broke, lactone bond might undergo hydrolysis. So, compound **6** was identified as 3-(7-(2,3-dihydroxy-3-methylbutoxyl)-6-hydroxy-5-benzofuranyl)-3-sulfopropanoic acid. Similarly, compound **5** was identified as 3-(7-(2-en-3-methylbutoxyl)-6-hydroxy-5-benzofuranyl)-3-sulfopropanoic acid. The fragment pathway is shown in Supplementary Figures SA8.

The structures of the markers are shown in Figure 2D. Detailed information of the markers is listed in Table 1. Sulfur-containing compounds only existed in sulfured Baizhi, among which, compound **3** showed a relatively high response in mass spectrometer. Therefore, we next purified compound **3** and obtained its structural information.

**TABLE 1:** Markers of sulfured and unsulfured Baizhi.

No	Primary ID	Formula	*p* (sulfured/unsulfured)	Factor of change	Average (sulfured)	Average (unsulfured)	Std.Dev (sulfured)	Std.Dev (unsulfured)	Assigned identity	Mass error/ppm
**1**	11.62_80.9624m/z	C_17_H_20_O_10_S	0.0000	>10 000	652886.00	0.01	6356.8047	0	3,4-dihydrobyakangelicin-4-sulfonic acid	−4.6
**2**	19.95_351.0524m/z	C_16_H_16_O_7_S	0.0003	>10 000	612783.00	0.01	>10 000	0	3,4-dihydroimperatorin-4- sulfonic acid	−4.3
**3**	13.34_386.0645n	C_16_H_18_O_9_S	0.0004	−343234400	1446250.00	0.00	>10 000	0	(4*R*,12*S*)-3,4-dihydrooxypeucedanin hydrate-4-sulfonic acid	−5.0
**2**	19.95_80.9625m/z	C_16_H_16_O_7_S	0.0003	−28730986	678774.00	−0.02	>10 000	0	3,4-dihydroimperatorin-4 sulfonic acid	−4.3
**4**	12.01_386.0646n	C_16_H_18_O_9_S	0.0022	−73907176	1112960.00	−0.02	>10 000	0	3,4-dihydrooxypeucedanin-3 hydrate sulfonic acid	−0.5
**3**	13.33_201.0167m/z	C_16_H_18_O_9_S	0.0001	5,839	1956890.00	335.17	>10 000	48.5538	(4*R*,12*S*)-3,4-dihydrooxypeucedanin hydrate-4-sulfonic acid	−0.5
**5**	16.84_299.9923m/z	C_16_H_18_O_8_S	0.0195	−88216400	2172000.00	−0.02	>10 000	0	3-(7-(2-en-3-methylbutoxyl)-6-hydroxy-5-benzofuranyl)-3-sulfopropanoic acid	−5.1
**6**	10.47_433.0798m/z	C_17_H_22_O_11_S	0.0286	−59905080	921477.00	−0.02	>10 000	0	3-(7-(2,3-dihydroxy-3-methylbutoxyl)-6-hydroxy-4-methoxyl-5-benzofuranyl)-3-sulfopropanoic acid	−3.7
**4**	12.01_201.0167m/z	C_16_H_18_O_9_S	0.0014	51	1607570.00	31341.20	>10 000	20065.5	3,4-dihydrooxypeucedanin-3 hydrate sulfonic acid	−0.5
**7**	20.72_330.2387n	C_18_H_34_O_5_	0.0133	14	258763.00	3590640.00	>10 000	1.36E+06	trihydroxy-octadecenoic acid	−0.8
**8**	31.02_383.3513m/z	C_24_H_48_O_3_	0.0110	12	67162.30	816443.00	>10 000	289813	Unknown	−1.2
**9**	14.32_540.1839n	C_25_H_32_O_13_	0.0000	27	29367.80	804740.00	>10 000	46651.8	(β-D-apiosyl)-β-D-glucosyl marmesin	−0.9
**10**	15.20_201.0165m/z	C_17_H_18_O_9_S	0.0049	4	1143890.00	311903.00	>10 000	72493.3	dihydrobyakangelicol sulfonic acid	0.8
**11**	13.53_540.1839n	C_25_H_32_O_13_	0.0009	18	48986.50	889418.00	>10 000	152424	iso-(β-D-apiosyl)-β-D-glucosyl marmesin	1.3
**12**	20.09–381.0631	C_17_H_18_O_8_S	0.0002	2,830	757924.00	267.84	>10 000	251.413	3,4-dihydrophellopterin-4-sulfonic acid	−3.4
**13**	24.52_296.2327n	C_31_H_47_NO_4_	0.0047	5	349326.00	1807520.00	>10 000	405150	unknown	−1.8
**14**	24.21_252.6581n	C_35_H_32_N_4_O_9_	0.0030	5	841348.00	178386.00	>10 000	146522	unknown	−0.6
**15**	0.59_342.1144n	C_12_H_22_O_11_	0.0243	2	2469570.00	3659350.00	>10 000	241987	maltose	−0.9
**16**	25.16_595.2891m/z	C_32_H_30_O_9_	0.0358	8	120474.00	956978.00	>10 000	433359	unknown	−0.4
**17**	26.19_311.1666m/z	unknown	0.0836	1	7185990.00	5816610.00	>10 000	411043	unknown	

### 3.2 Structural Analysis of Compounds 1 and 3

**FIGURE 3:**
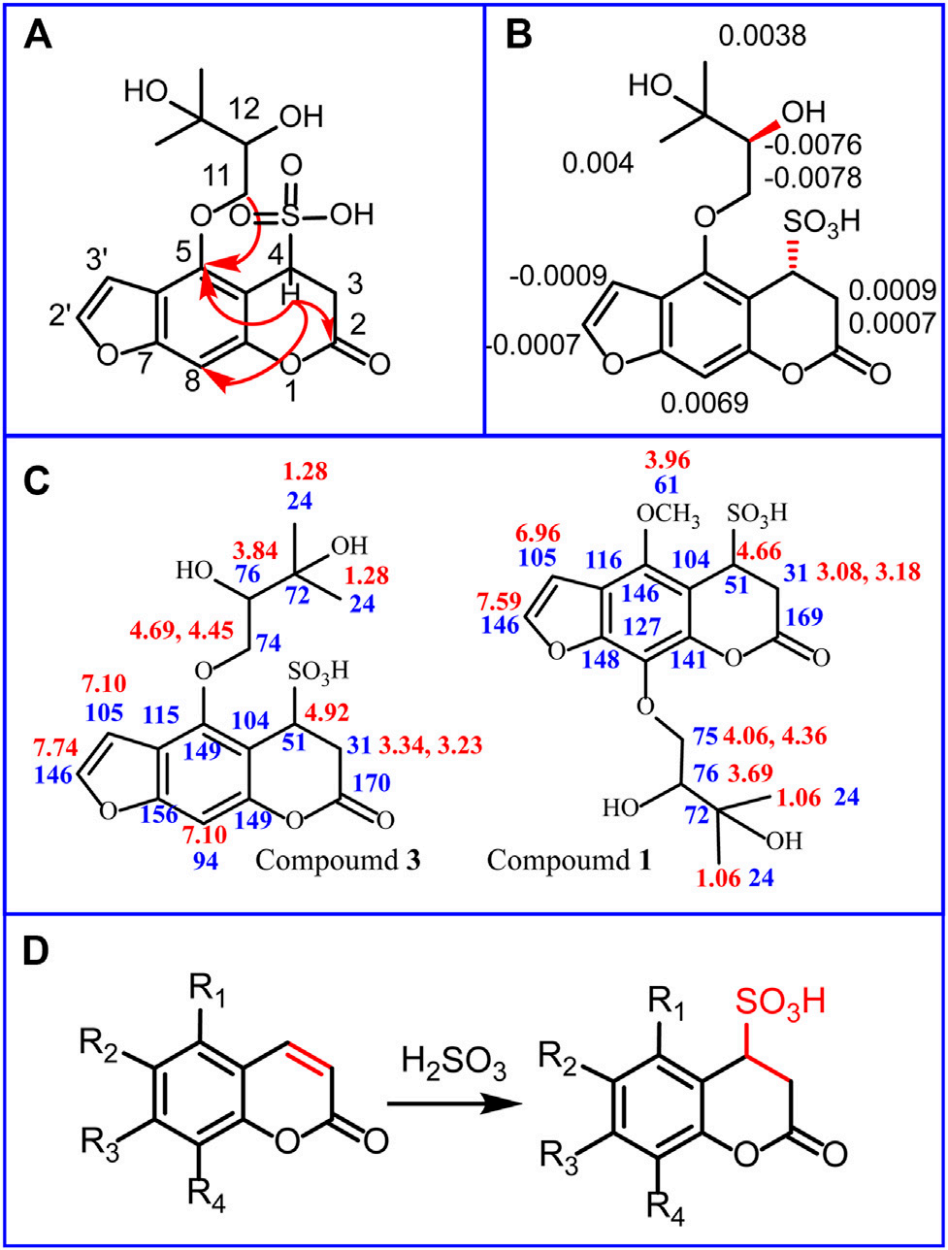
Structure determination of compound 1, 3 and chemical transformation mechanism in sulfuring process. Key heteronuclear multiple bond correlations (HMBCs) of compound **3 (A)**; Δδ values (Δδ = δ_S_ − δ_R_) obtained from the methyl-α-trifluoromethylphenyl lactic acid (MTPA) esters of compound **3 (B)**; ^1^H NMR (marked red) and ^13^C NMR (marked blue) values of compound 1 and compound **3 (C)**; Chemical transformation mechanism in sulfuring **(D)**.

Compound **3** was obtained as a yellow powder. Its molecular formula, C_16_H_18_O_9_S, was established using UPLC-Q-TOF-MS with *m/z* 385.0591 [M-H]^−^ (calcd 385.0587), *m/z* 771.1204 [2M-H]−(calcd 771.1212), and *m/z* 793.1116 [2M-Na+2H]^−^ (calcd 793.1084). Compared with (*R*)-heraclenol and OXH-N (C_16_H_16_O_6_) in unsulfured Baizhi, there was one additional H_2_SO_3_ in compound **3**. Compound **3** was predicted to be a sulfite derivative of (*R*)-heraclenol or OXH-N. In the ^1^H NMR (600 MHz, D_2_O) spectrum of compound **3**, ^1^H NMR (600 MHz, D2O) values comprising δ 7.75 (s, 1H), 7.10 (s, 2H), 4.92 (s, 1H), 4.69 (d, J = 10.2 Hz, 1H), 4.45 (t, J = 8.6 Hz, 1H), 3.84 (m, 1H), 3.34 (d, J = 17.4 Hz, 1H), 3.23 (d, J = 17.4 Hz, 1H) and 1.28 (d, J = 11.6 Hz, 6H) were observed. These data were consistent with the spectrum of oxypeucedanin hydrate (Yu et al., 2010). However, δ_H_ 4.92 (H, s, H-4) and 3.29 (H, d, J = 17.4 HZ, H-3) were lower in the spectrum of compound **3** than in that of oxypeucedanin hydrate because the single bond between C-3 and C-4 changed into a double bond and the sulfur atom was directly linked to C-4. Additionally, δ_H_ 4.92 (H, s, H-4) suggested a sulfonate group connected to C-4. ^13^C NMR (125 MHz, D_2_O) values are listed in Supplementary Table SA1. Correlations between H-3 and C-2/C-4/C-10 and H-4 and C-2/C-3/C-5/C-9/C-10 were observed, suggesting that the sulfonate moiety was connected to C-4. Moreover, the connections between H-11 and C-5/C-12/C-13, H-12 and C-13/C-14/-C15, H-14 and C-12/C-13/C-15, H-15 and C-12/C-13/C-14 indicated that 2,3-dihydroxy-3-methylbutoxy was connected to C-5. The key HMBC correlations of compound **3** are shown in Figure 3A. Detailed high-resolution electrospray ionization MS spectra and related fragments, related HMBC spectra, HSQC spectra, ^1^H NMR spectra, and ^13^C NMR spectra of compound **3** are shown in Supplementary Figures SA1, 9, 10–12, respectively.

To determine the stereostructure of compound **3,** the absolute configuration at the C-12 position was confirmed using Mosher’s method as described in *Structural Analysis of Compounds 1 and 3*. Comparison of the ^1^H NMR data for the *R*-(**3**)- and *S*-(**3**)-MTPA esters of **3** in CDCl_3_ (Figure 3B) showed that C-12 had an (*S*)-absolute configuration. (Supplementary Figure SA13). The electronic circular dichroism data were then calculated, and the low-energy conformers of (4*R*,12*S*)-**3** and (4*S*,12*S*)-**3** are presented in Supplementary Tables SA2, 3. Compound **3** was named (4*R*,12*S*)-3,4-dihydrooxypeucedanin hydrate-4-sulfonic acid (OXH-S).

Compound **1** was obtained as a brown powder. Its molecular formula, C_17_H_20_O_10_S, was established using UPLC-Q-TOF-MS with *m/z* 415.0691 [M-H]^-^ (calcd 415.0699), *m/z* 831.1523 [2M-H]^-^ (calcd 831.1476), and *m/z* 853.1298 [2M-Na+2H]^-^ (calcd 853.1296). Compared with byakangelicin (C_17_H_18_O_7_) in unsulfured Baizhi, there was one additional H_2_SO_3_ in compound **1**. Compound **3** was predicted to be a sulfite derivative of byakangelicin. In the NMR spectrum of compound **1**, ^1^H NMR (500 MHz, D2O) values of δ 7.59 (dd, J = 2.0, 1.5 Hz, 1H), 6.96 (d, J = 1.8 Hz, H), 4.66 (s, H), 4.36 (ddd, J = 11.1, 9.0, 2.3 Hz, H), 4.06 (dt, J = 11.0, 8.5 Hz, H), 3.69 (m, H), 3.18 (dd, J = 17.3, 1.6 Hz, H), 3.08 (ddd, J = 17.3, 6.4, 3.9 Hz, H) and 1.06 (s, 6H) were observed. These data were in agreement with the spectrum of compound **3**. However, compound **1** had δ_H_ 3.96 (s, 3H), which was not present in compound **3**, indicating an extra methyl group in compound **1**. Compared with compound **3,** δ_H_ 7.10 (s, 2H) was missing**,** indicating that the extra methyl group was connected to C-5. ^1^H NMR and ^13^C NMR of compound **1** and compound **3** are shown in Figure 3C. Moreover, in HMBC spectra, the connections between H-16 and C-5 indicated that methoxy was connected to C-5. The connections between H-11 and C-8/C-12/C-13 indicated that 2,3-dihydroxy-3-methylbutoxy was connected to C-8. The other connections were the same as those in compound **3**. Detailed high-resolution electrospray ionization MS spectra and related fragments, related HMBC spectra, HSQC spectra, ^1^H NMR spectra, and ^13^C NMR spectra of compound **1** are shown in Supplementary Figures SA1 and SA15–18, respectively. Compound **1** was named 3,4-dihydrobyakangelicin-4-sulfonic acid.

These data demonstrated that transformation of coumarins into dihydrocoumarin sulfonic acid was a reliable result (Figure 3D). The main reaction in the sulfuring process was inferred as an addition reaction between coumarins and sulfinic acid. The double bond between C-2 and C-3 was disrupted during the reaction. The addition of a sulfonic acid group can increase the polarity of coumarins. This might affect the absorption, transformation, and excretion of coumarins in the body. The sulfuring process can alter the abundance and content of coumarins, which may also influence their efficacy and cytotoxicity. Therefore, we next evaluated whether these changes were beneficial or harmful in terms of cytotoxicity and anti-inflammatory activity.

### 3.3 Cytotoxicity Evaluation

**FIGURE 4:**
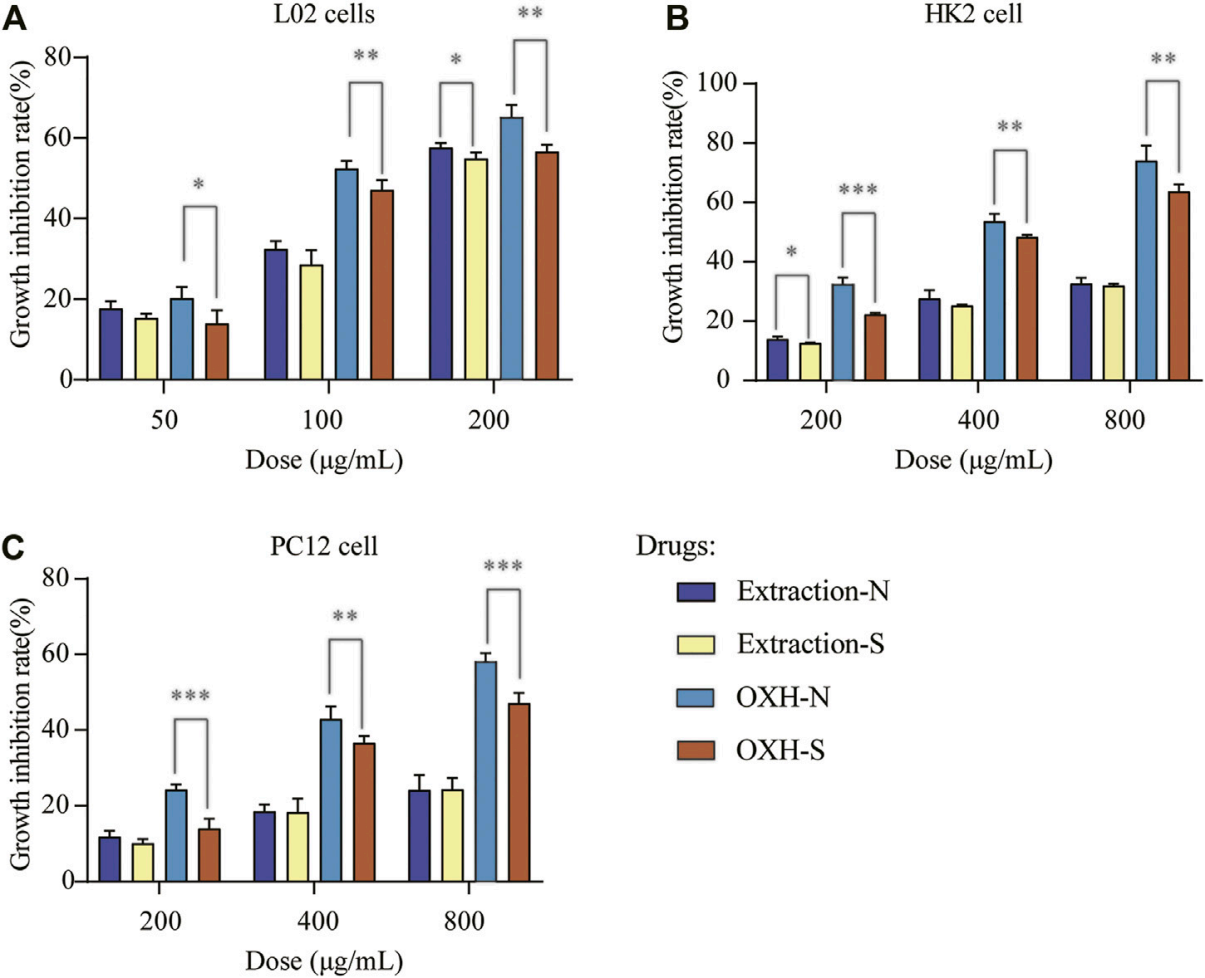
Cell growth inhibition rate with each drug based on **(A)** L02 cells, **(B)** HK2 cells, and **(C)** PC12 cells (*n* = 3; *p < 0.01, **p < 0.05, ***p < 0.001).

The most important concern during sulfuring is safety, as many researchers have suggested that the sulfuring process leads to health risks (Kang et al., 2017; Zhou et al., 2019). Therefore, the cytotoxicity of OXH-N, OXH-S, extraction-N, and extraction-S towards L02, HK2, and PC12 cells was evaluated. These compounds showed dose-dependent toxicity towards all cells evaluated at doses of 50–200 mg/L (Figure 4). Compared with OXH-N, OXH-S was less toxic to each cell strain at the same dosage. The results of the cytotoxicity evaluation *t-*test are shown in Supplementary Table SA4. The original data was shown in Supplementary Data Sheet 3. Compared with extraction-N, extraction-S was less toxic to each cell strain, but there was no significant difference at the same dosage. Our results were consistent with those of a previous study (Lu, 2013) and indicated that sulfured Baizhi is less toxic than unsulfured Baizhi. The reason might be that dihydrocoumarin sulfonic acids (with relatively weaker lipophilicity) are more difficult to be absorbed by cells and dihydrocoumarin sulfonic acids were less toxic than coumarins. However, further studies on animals should be conducted to determine whether sulfur fumigation can be applied to Baizhi.

### 3.4 Anti-Inflammatory Effects

#### 3.4.1 MTC of Extraction-N and OXH-N in Zebrafish Inflammation Model

Extraction-N and extraction-S had similar chemical compositions and OXH-N and OXH-S had similar structures. Therefore, prior to testing the anti-inflammatory activity of extraction-N, extraction-S, OXH-N, and OXH-S, the toxicity of extraction-N and OXH-N was examined. At 2 h after exposure to extraction-N and OXH-N, no zebrafish died with a concentration range of 125–500 μg/ml. However, at concentrations of 1,000 and 2000 μg/ml, the drugs precipitated. Accordingly, the MTC was 500 μg/ml. Based on these results, 55.6, 167, and 500 μg/ml were selected for subsequent experiments.

#### 3.4.2 Evaluation of Anti-Inflammatory Effects of Extraction-N, Extraction-S, OXH-N, and OXH-S Using a Zebrafish Inflammation Model

An LPS-induced zebrafish inflammation model was used to evaluate the anti-inflammatory effects of the drugs. Many fluorescent neutrophils were recruited at the inflammation site in the LPS-induced zebrafish. The average number of fluorescent neutrophils at the inflammatory site of the LPS-induced zebrafish model was 17.9 ± 1.26, whereas that in the blank control group was 4.8 ± 0.66. Compared with that in the blank control group, inflammatory regression was 45% in the model group (*p* < 0.01). Therefore, this model was deemed suitable for evaluating zebrafish inflammation.

**FIGURE 5:**
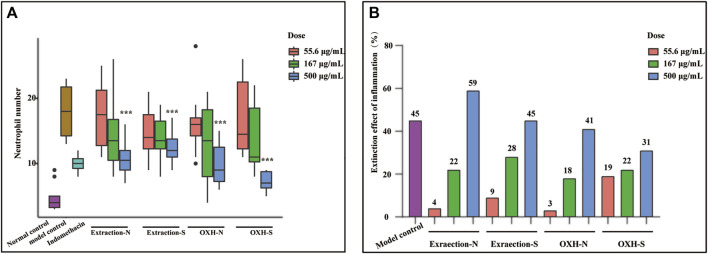
Anti-inflammatory effect evaluation. **(A)** number of neutrophils in zebrafish yolk sac (*n* = 10; compared with that in model control group, *p < 0.01, **p < 0.05, ***p < 0.001); **(B)** inflammatory regression in each group (*n* = 10).

The mean number of fluorescent neutrophils in the inflammatory sites of zebrafish and inflammatory regressions treated with different drugs at different dosages is shown in Figure 5. Compared with that in the model group, extraction-N, extraction-S, OXH-S, and OXH significantly decreased the number of neutrophils at a concentration of 500 μg/kg, and the effect of each drug was dose-dependent. However, except for the 500 μg/ml dosage, there was no significant difference between OXH-N and OXH-S or between extraction-N and extraction-S at the same dosage (*t*-test of inflammatory regression shown in Supplementary Table SA5). The original data was shown in Supplementary Data Sheet 2. This indicates that there is no significant difference in the anti-inflammatory effects of unsulfured and sulfured Baizhi.

### 3.5 Stability of Markers and Sulfur Dioxide Residue During Boiling

**FIGURE 6:**
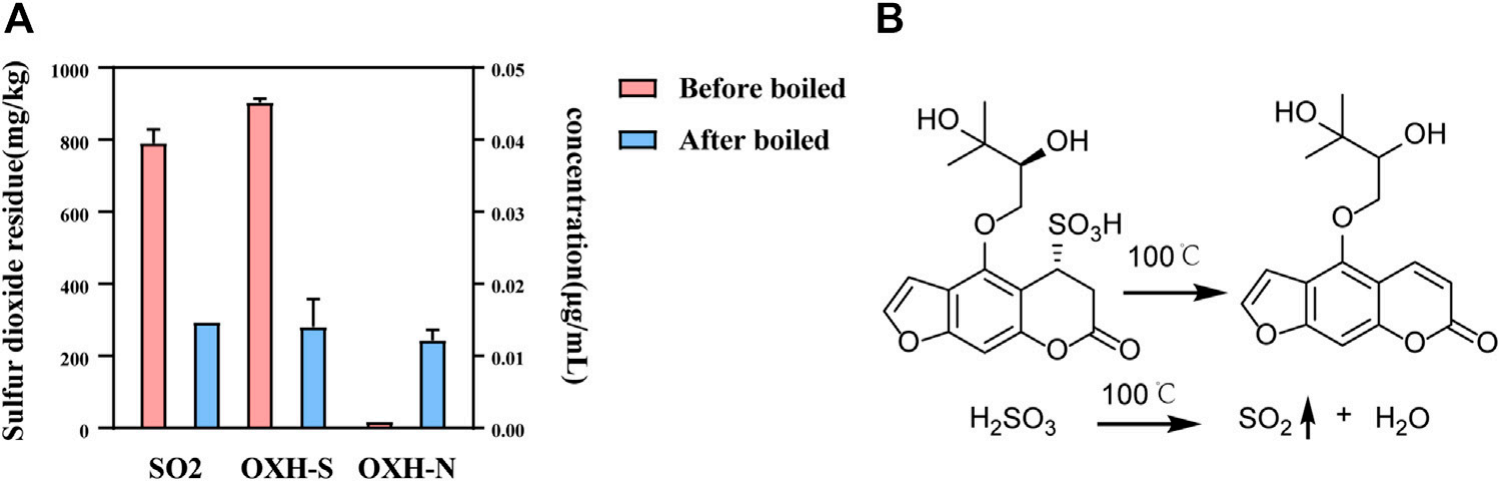
Stability of OXH-S and sulfur dioxide residue during the boiling process. **(A)** Content of OXH-S and sulfur dioxide residue (*n* = 3); **(B)** Chemical transformation of OXH-S and sulfite.

Whether used as a spice or TCM, it is necessary to boil Baizhi. Therefore, the stability of OXH-S during boiling was tested. After 1 h of boiling, the concentration of OXH-S decreased by 75%, and OXH-N emerged (Figure 6A), indicating that OXH-S is unstable and transforms into OXH-N during boiling. To ensure the repeatability of the experiment, the boiling temperature was set at 100 °C for 1 h. The sulfur dioxide residue level was 767 mg/kg before boiling but decreased by 62%–293 mg/kg after boiling for 1 h (Figure 6A). The chemical transformation of OXH-S and sulfite is shown in Figure 6B. In practice, the cooking temperature is higher than that used in our study. Therefore, the transformation rate might be greater than 75% during boiling. Our results indicate that decocting at 100°C for 1 h reduces the effect of sulfur fumigation on the chemical composition of Baizhi to some extent. Therefore, appropriate boiling could decrease the health risk of sulfur dioxide residues.

## 4 Conclusion

This study demonstrated that sulfur fumigation of Baizhi influences its chemical composition, leading to transformation of the coumarins into dihydrocoumarin sulfonic acids, and conversion of (4*R*,12*S*)-3,4-dihydrooxypeucedanin hydrate-4-sulfonic acid into oxypeucedanin hydrate during boiling. Additionally, sulfur fumigation (with a sulfur dioxide residue of 767 mg/kg) was found to decrease cytotoxicity without adversely affecting the anti-inflammatory properties of Baizhi. However, further studies are required to verify these results and conclusively depict the effects of sulfur fumigation on Baizhi. Interestingly, we also observed that combining sulfite with other compounds might improve their water solubility. For example, andrographolide total ester sulfonate injection (Xiyanping Zhusheye) and menadione sodium bisulfite, which are treated with sulfite, have been used clinically and are recorded in the Chinese Pharmacopeia (Zhao et al., 2017). Thus, sulfur fumigation might not always have adverse effects on the quality and therapeutic values of TCMs.

This study had some limitations. Although the anti-inflammatory effect of Baizhi is not the only pharmacological effect of this TCM, it is considered the most important attribute (Chen et al., 2015). Nonetheless, owing to the limited amount of the isolated compounds (300 mg), the anti-inflammatory effect could only be tested on zebrafish, and hence, studies on other animal models, such as rats and monkeys, should be performed for more reliable results. Moreover, toxicity experiments were conducted on cells and not on animal models; thus, further studies evaluating pharmacological effects and toxicity using animals are required. Regardless of these challenges, the present strategy provides a strong foundation for studying the safety, use, structure–activity relationship, and quality control of Baizhi, as well as the mechanisms of chemical transformation of other sulfured TCMs.

## Data Availability

The original contributions presented in the study are included in the article/Supplementary Material, further inquiries can be directed to the corresponding authors.

## Abbreviations

AAALAC, Association for assessment and accreditation of laboratory animal care; dpf, Days post-fertilization; EGFP, Enhanced green fluorescent protein; Extraction-N, Extraction of unsulfured Baizhi; Extraction-S, Extraction of sulfured Baizhi; FBS, Fetal bovine serum; HMBC, Heteronuclear multiple bond correlation; HPLC, High performance liquid chromatography; HSQC, Heteronuclear single quantum coherence; LPS, Lipopolysaccharide; MPX, Myeloid-specific peroxidase; MTC, Maximum tolerated concentration; MTPA, Methyl-a-trifluoromethylphenyl lactic acid; NMR, Nuclear magnetic resonance; OXH-N, Oxypeucedanin hydrate; OXH-S, (4R,12S)-3,4-dihydrooxypeucedanin hydrate-4-sulfonic acid; UPLC-Q-TOF-MS, Ultra-performance liquid chromatography coupled with quadrupole time-of-flight-mass spectrometry.
