# Supplementary material for: Practical Protocol for Comprehensively Evaluating Sulfur-Fumigation of Baizhi Based on Metabolomics, Pharmacology, and Cytotoxicity
Source: Front Pharmacol. 2022 Jan 25;12:799504. doi: 10.3389/fphar.2021.799504 (PMC8822044; doi:10.3389/fphar.2021.799504)
Supplement: Supplementary file 2 [file DataSheet1.docx]

# Supplementary Material


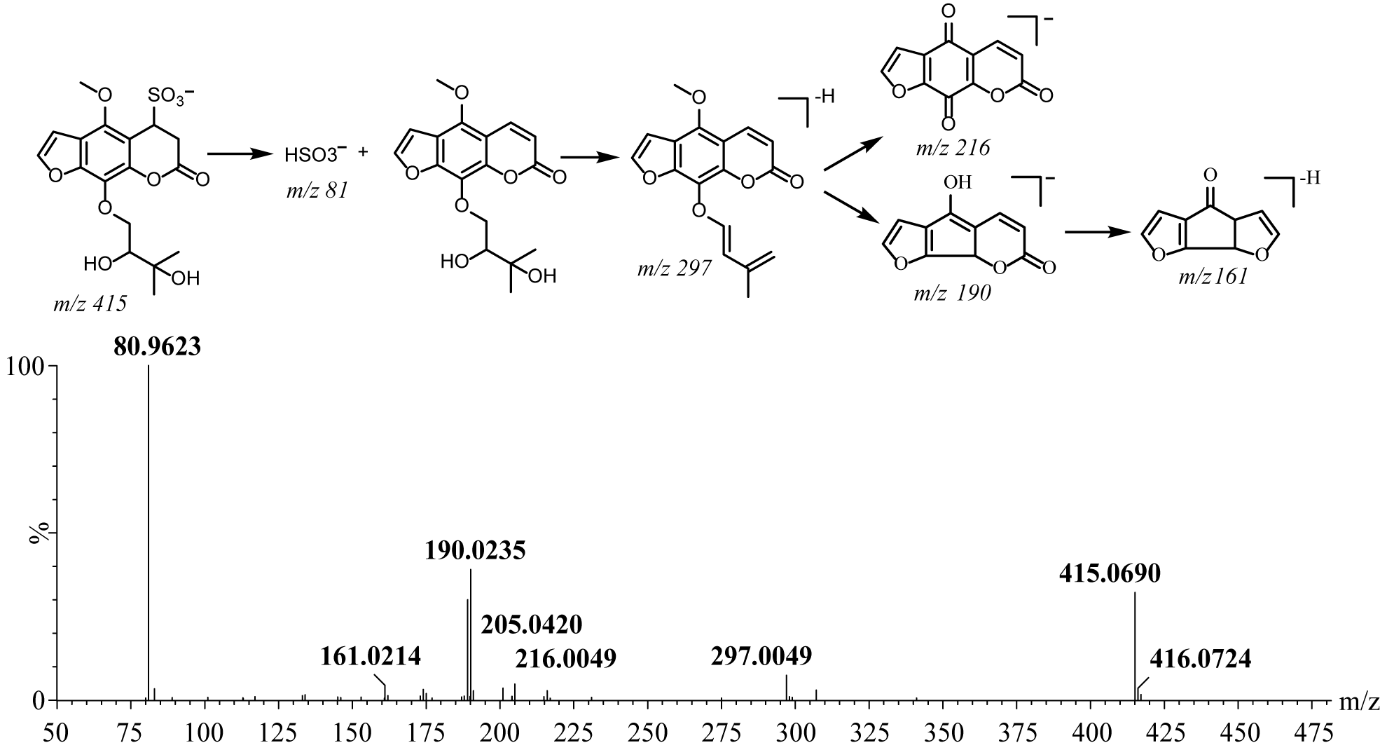


Fig. A1 High-resolution mass spectrum and related fragment ions of compound **1**

**
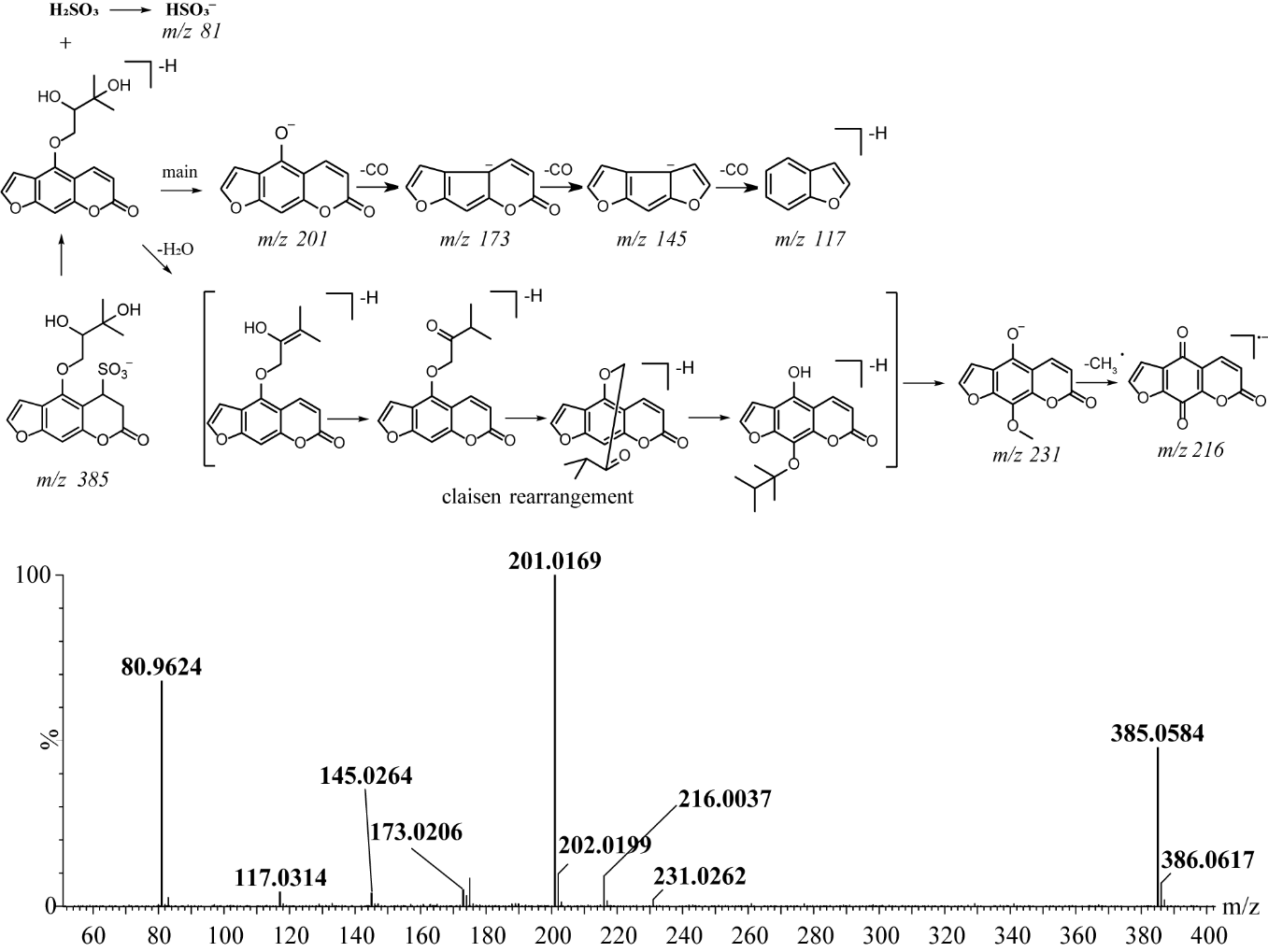
**

**Fig. A2** High-resolution mass spectrum and related fragment ions of compound **3**

**
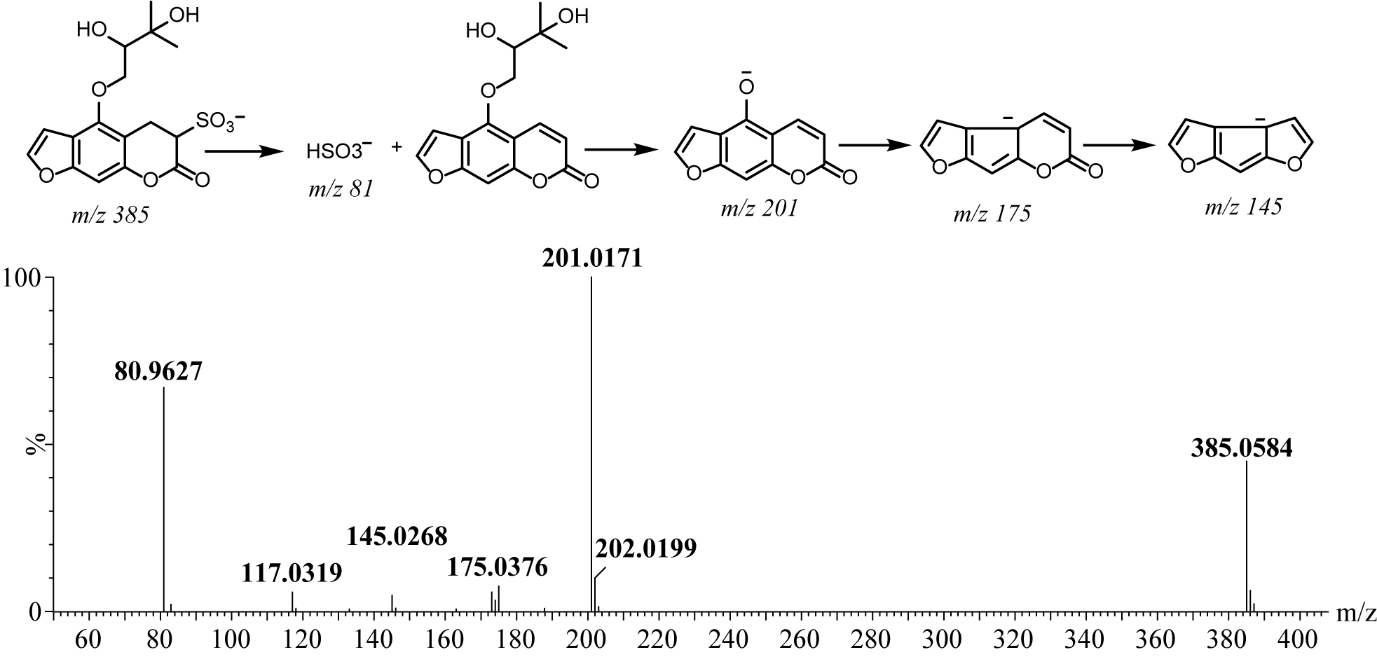
**

Fig. A3 High-resolution mass spectrum and related fragment ions of compound **4**

**
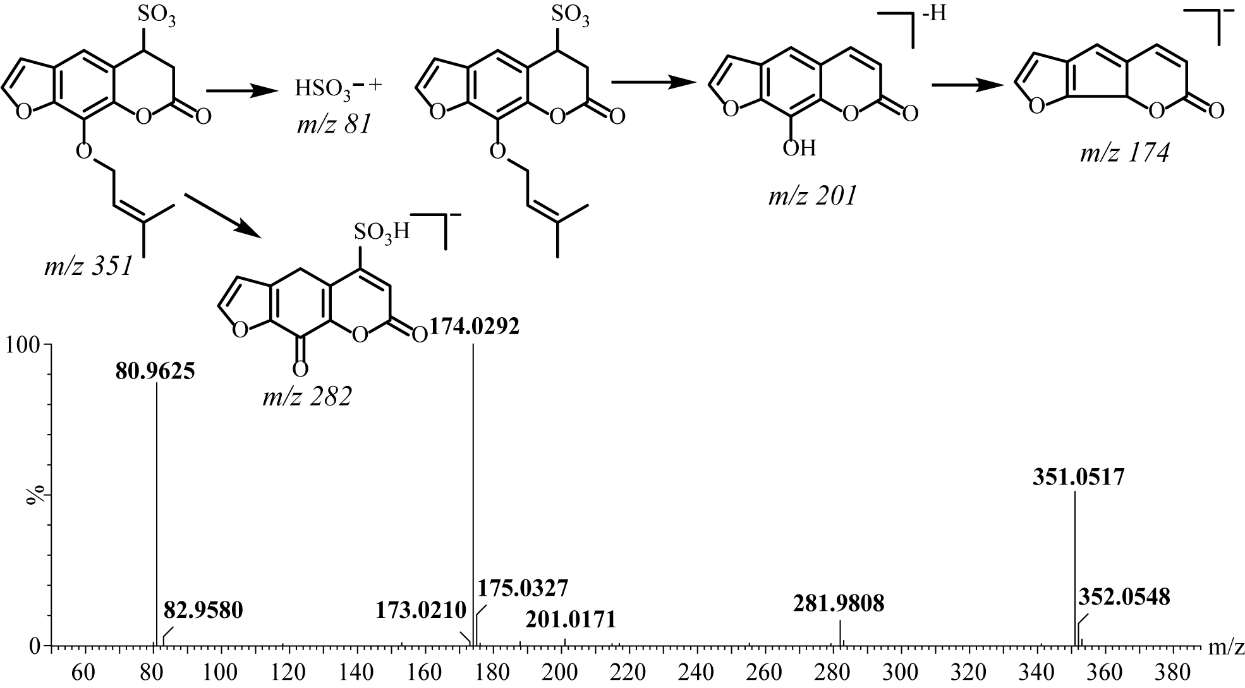
**

Fig. A4 High-resolution mass spectrum and related fragment ions of compound **2**

**
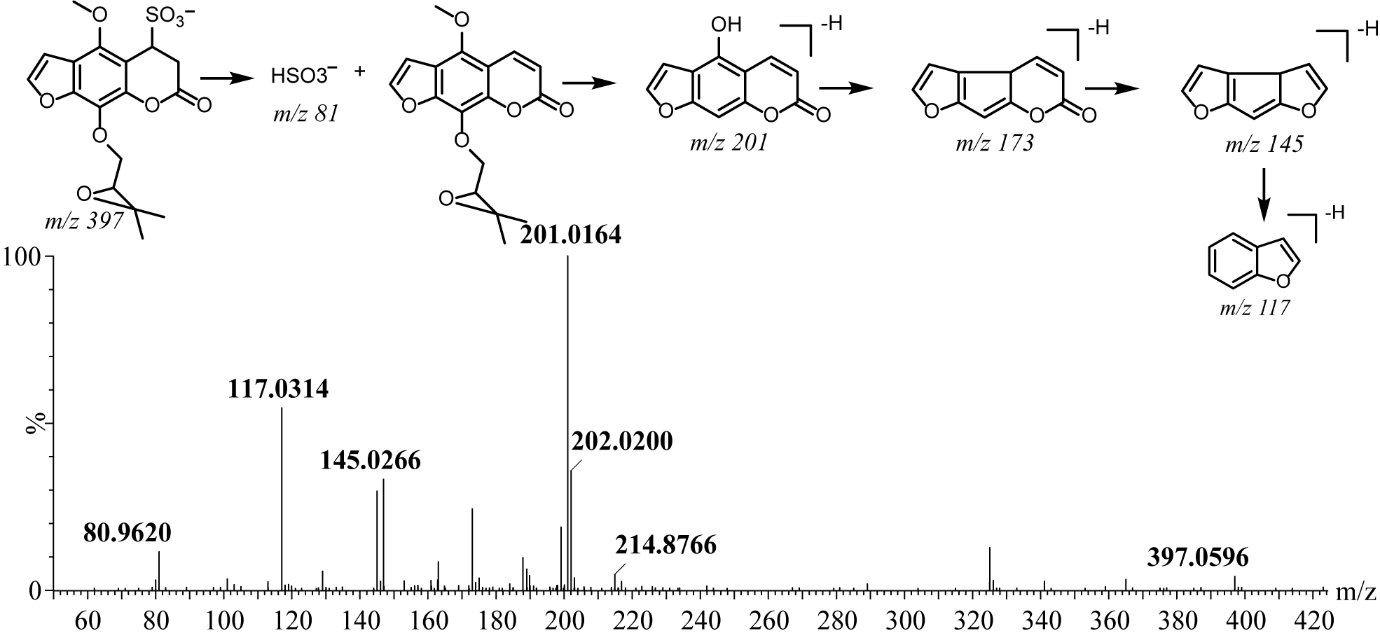
**

Fig. A5 High-resolution mass spectrum and related fragment ions of compound **10**

**
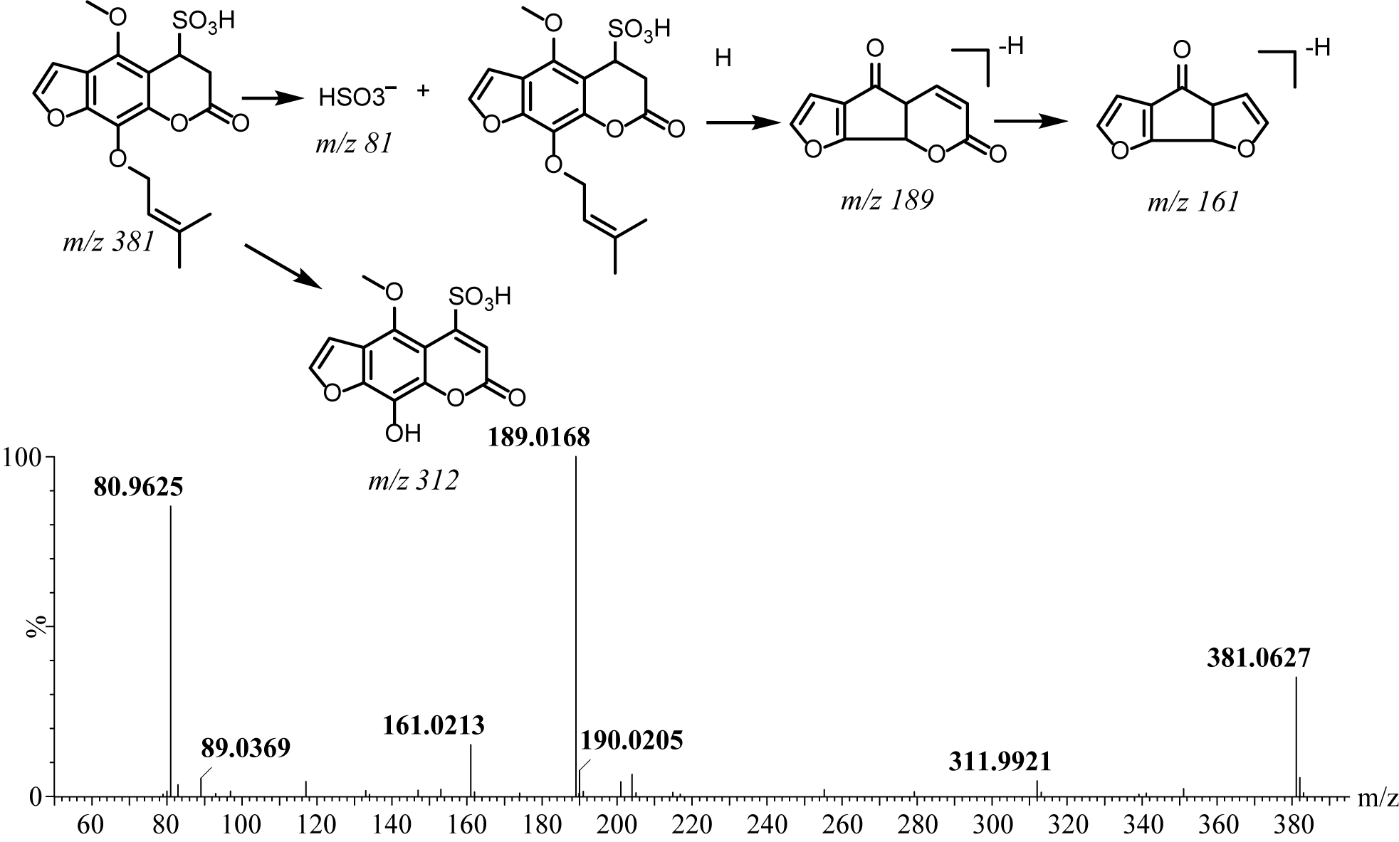
**

Fig. A6 High-resolution mass spectrum and related fragment ions of compound **12**

**
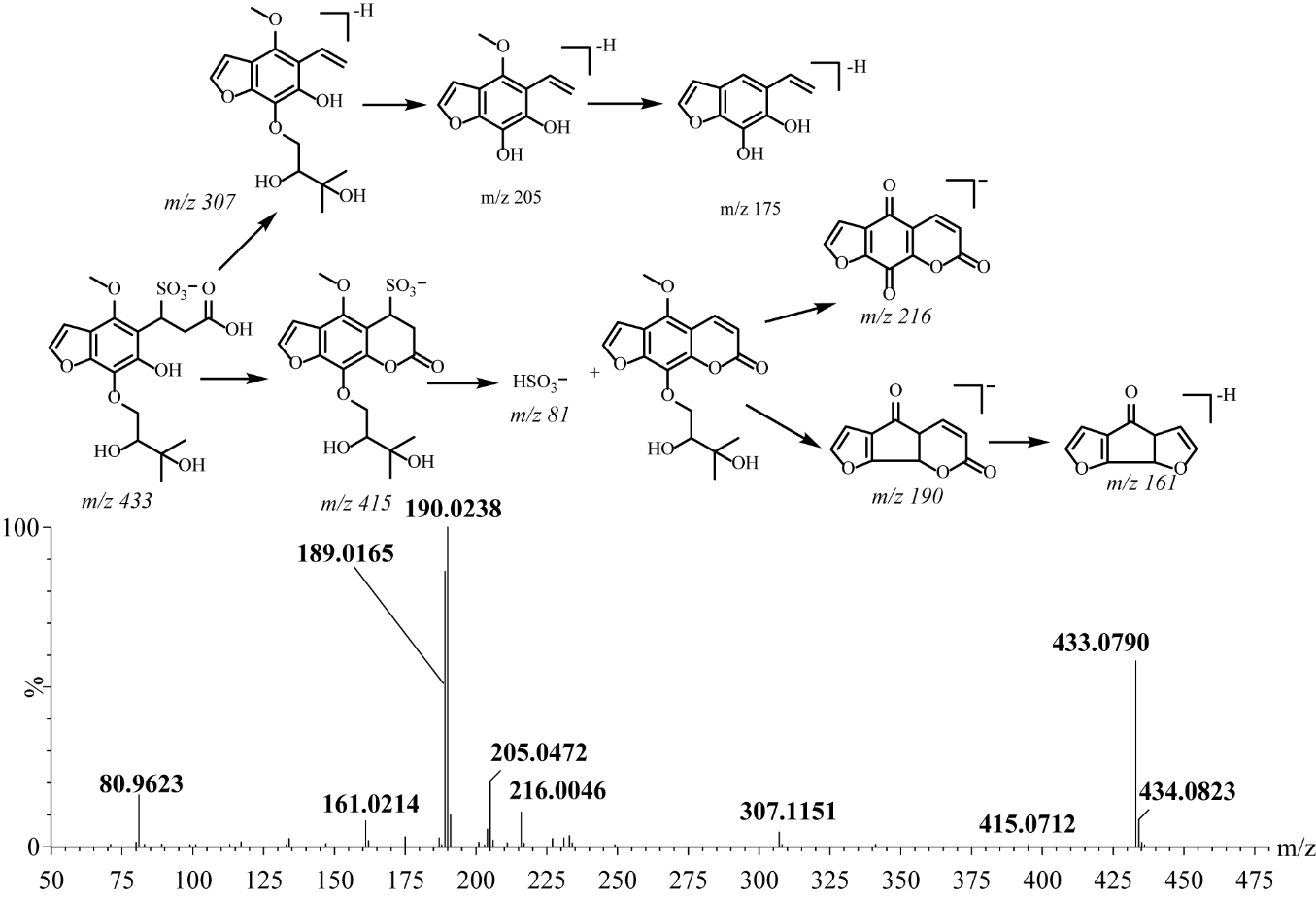
**

Fig. A7 High-resolution mass spectrum and related fragment ions of compound **6**

**
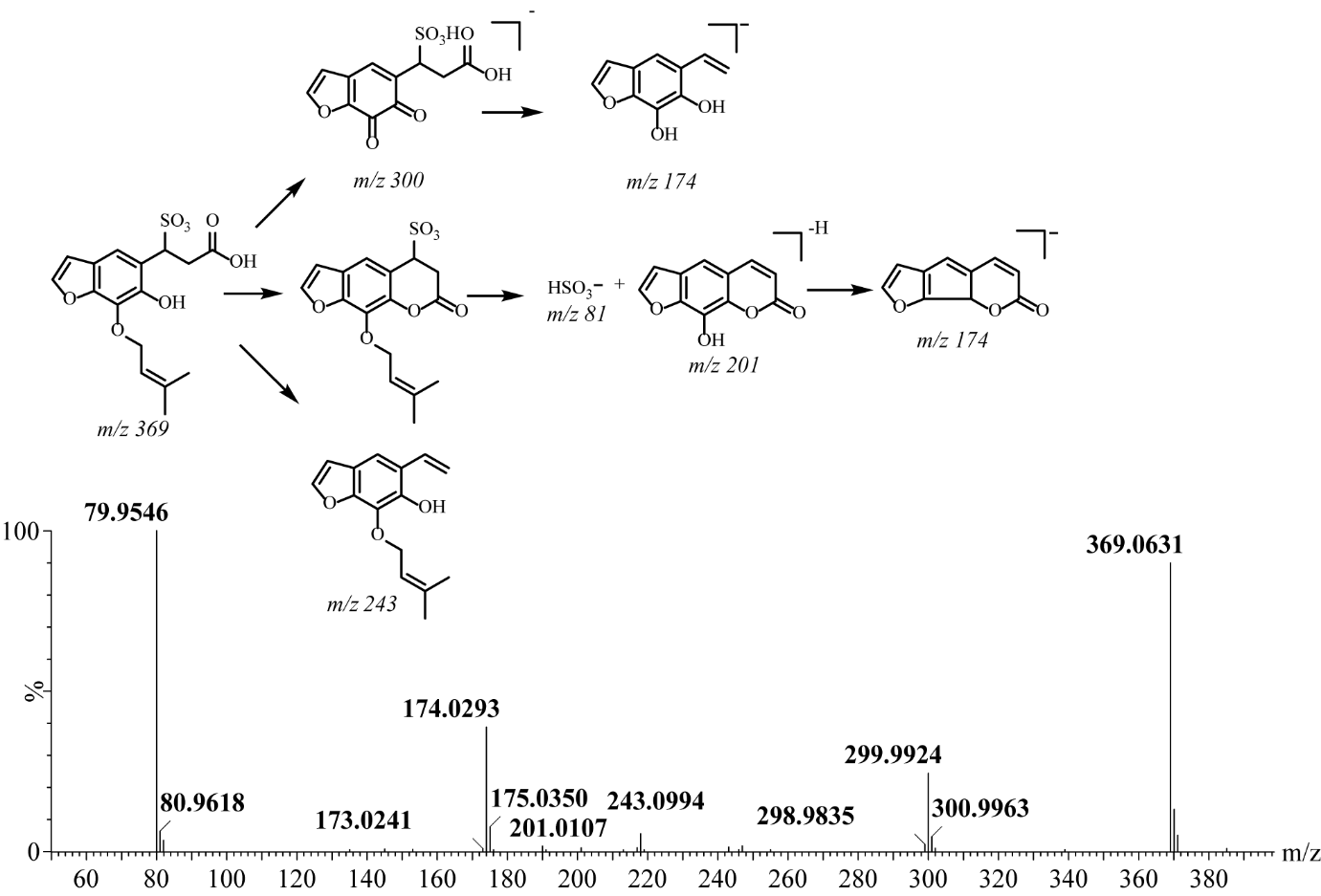
**

Fig. A8 High-resolution mass spectrum and related fragment ions of compound **5**


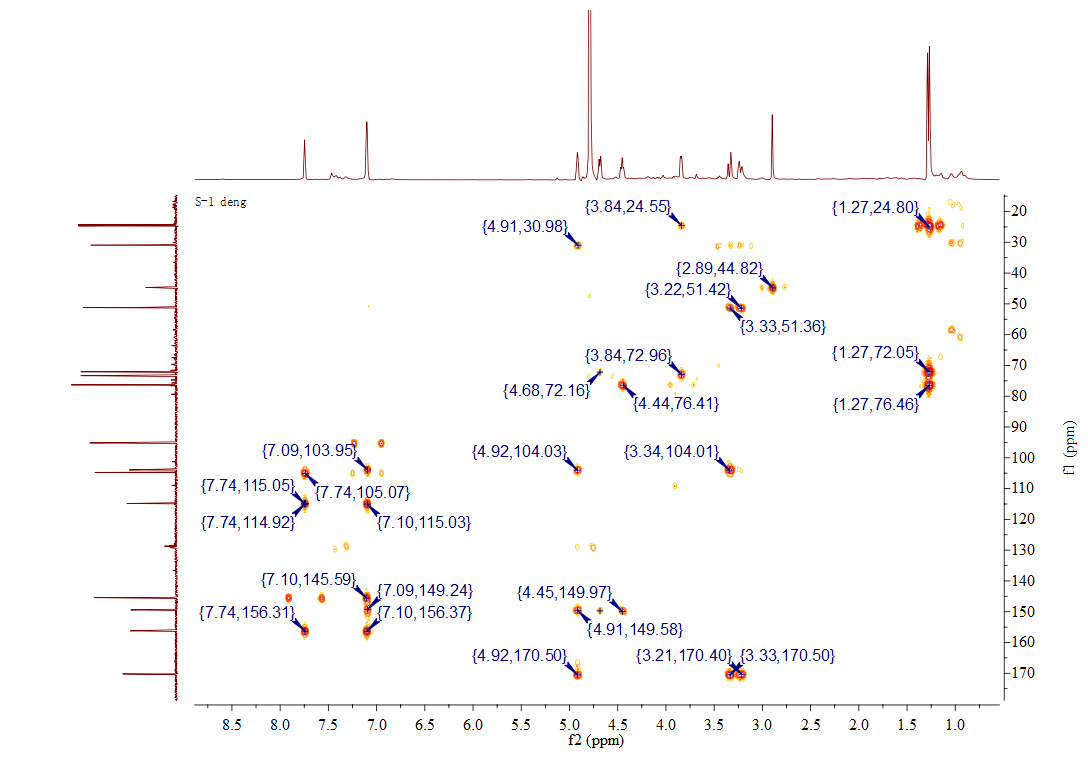


**Fig. A9** HMBC spectra of compound **3**

**
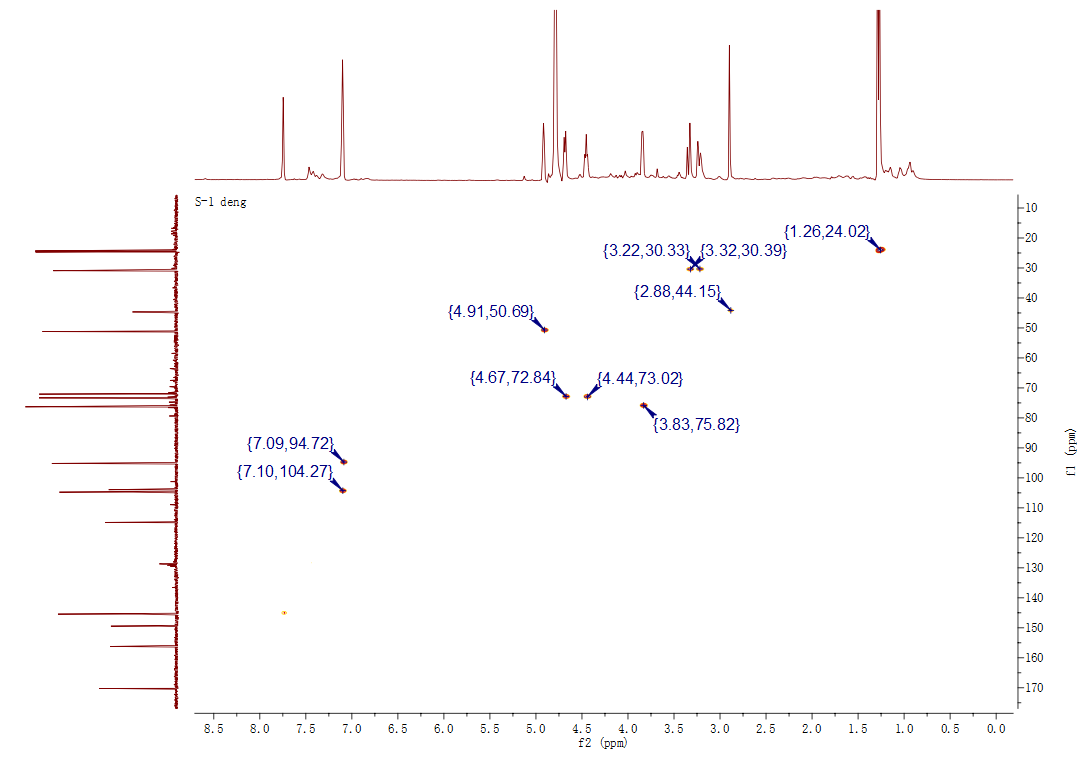
**

**Fig. A10** HSQC spectra of compound **3**

**
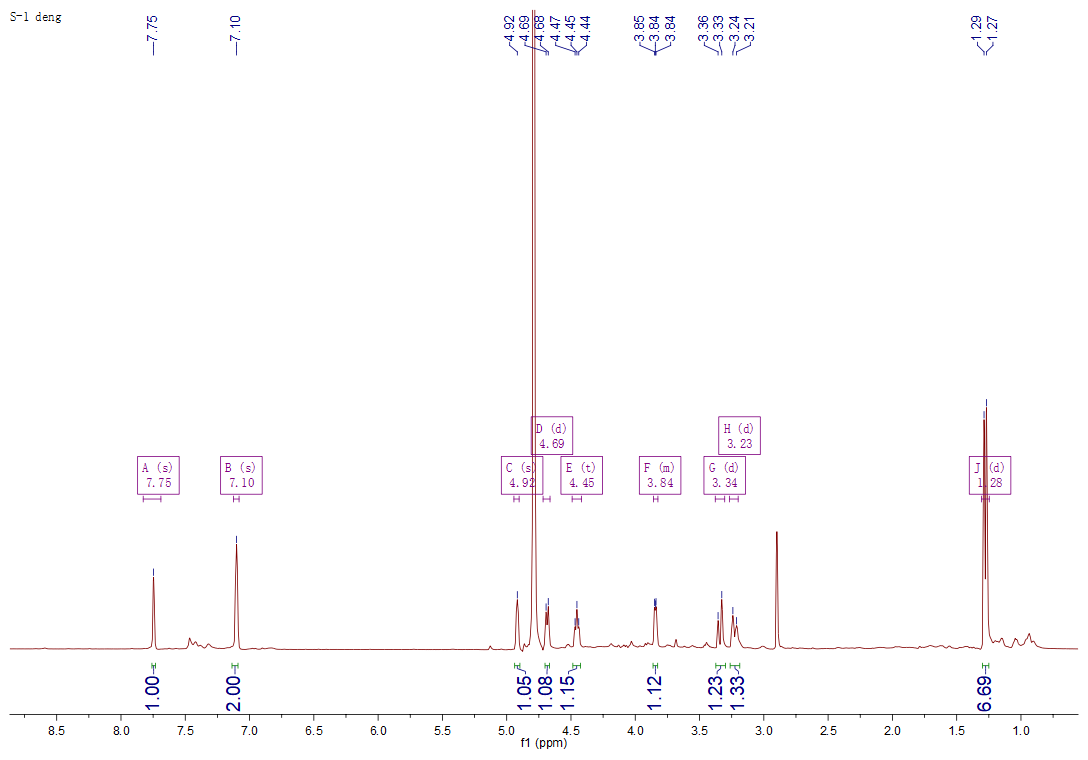
**

**Fig. A11** ^1^H NMR (600 MHz, D_2_O) spectra of compound **3**


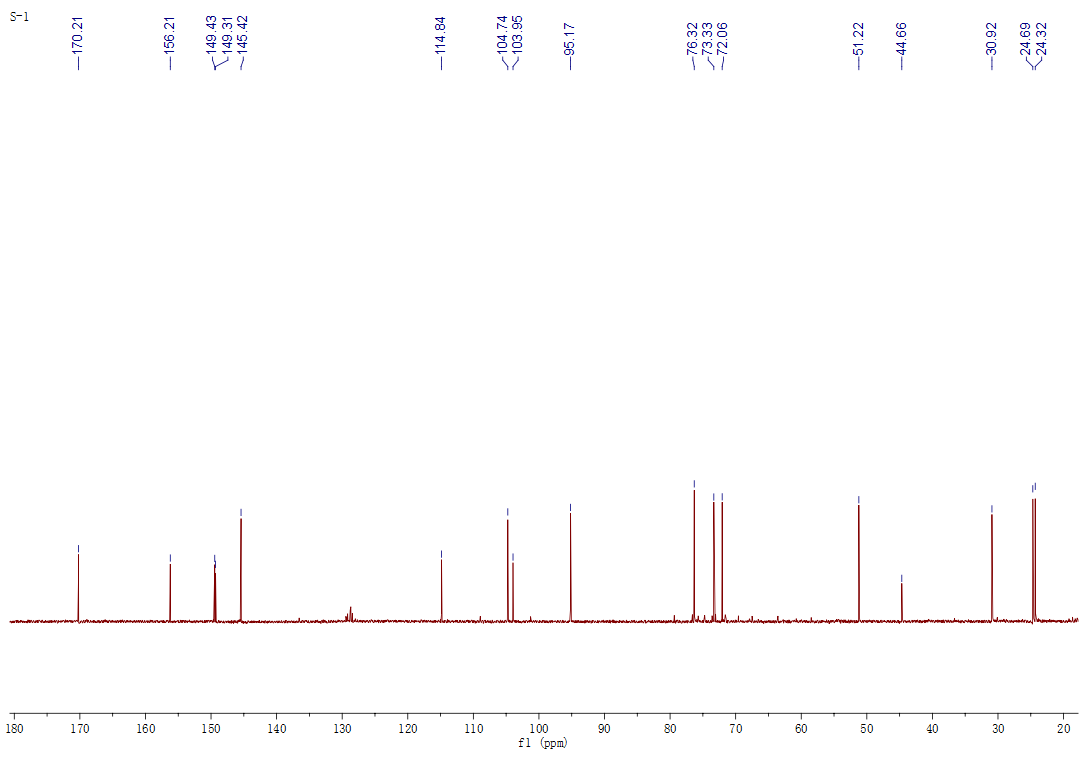


**Fig. A12** ^13^C NMR (125 MHz, D_2_O) spectra of compound **3**

**
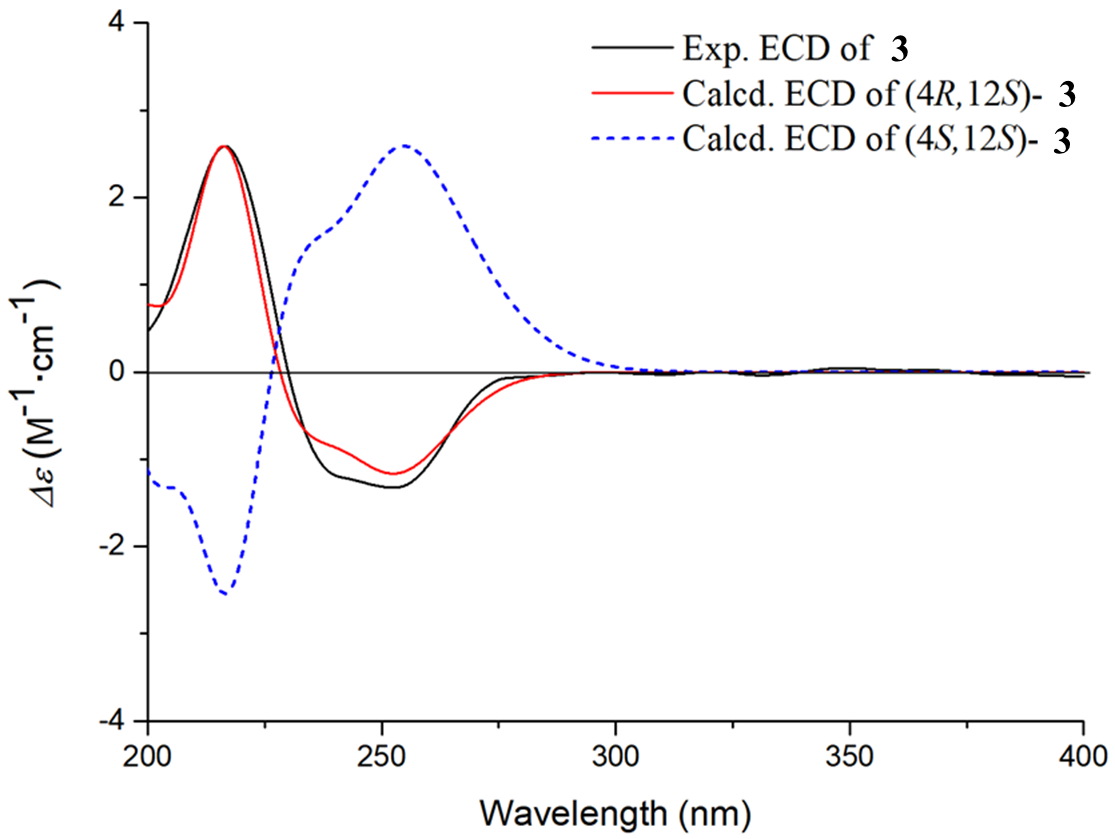
**

**Fig. A13** Experimental and calculated ECD spectra of **3** in MeOH

**
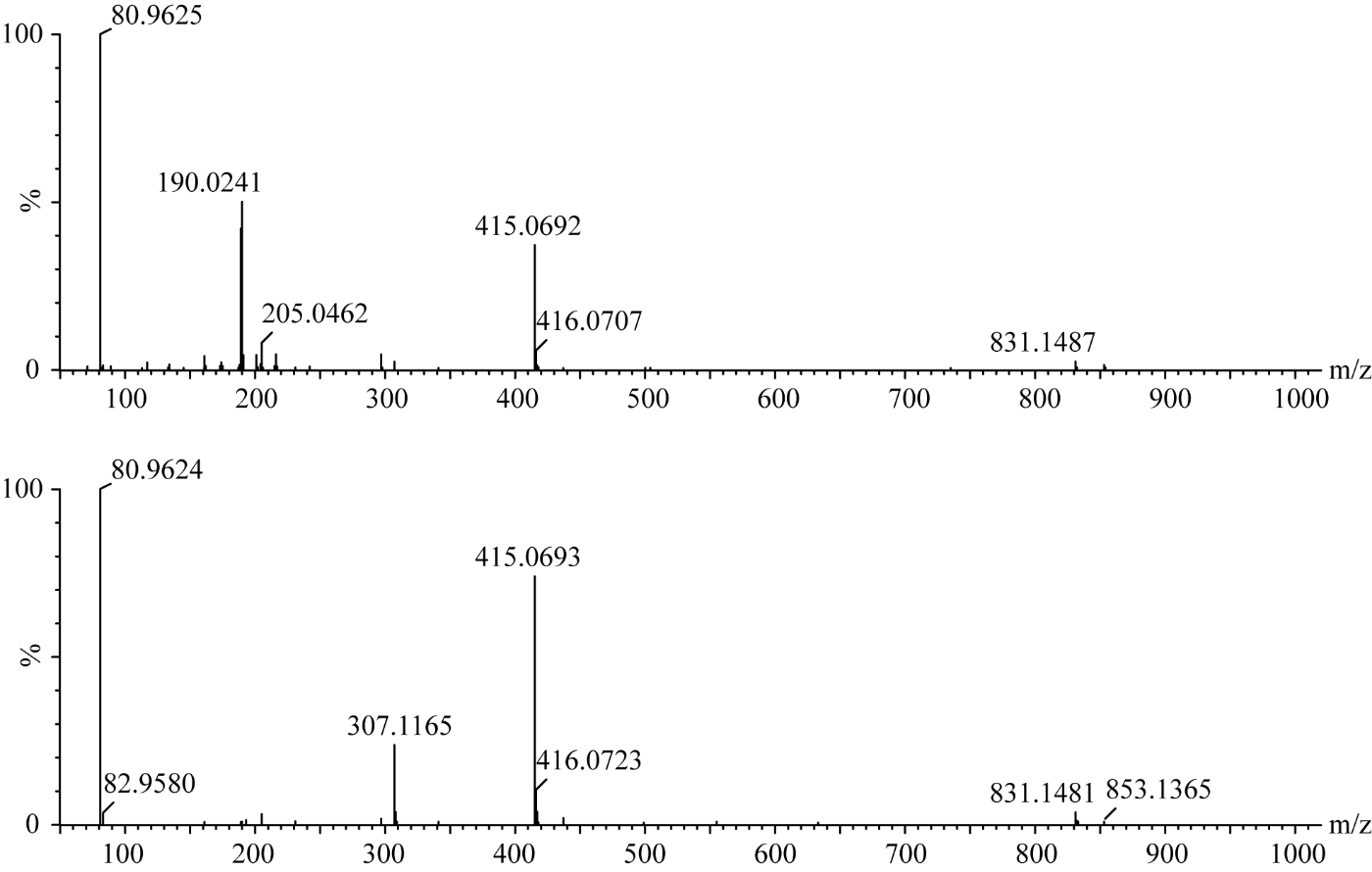
**

**Fig. A14** High-resolution mass spectrum of compound **1**


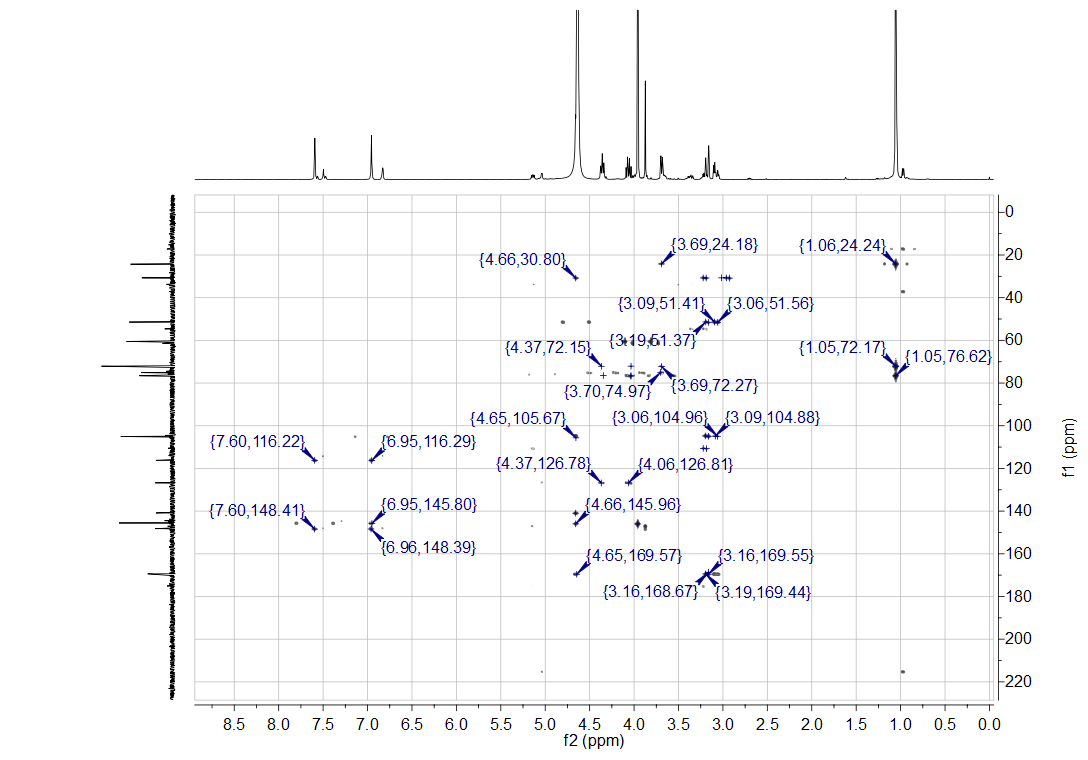


**Fig. A15** HMBC spectra of compound **1**

**
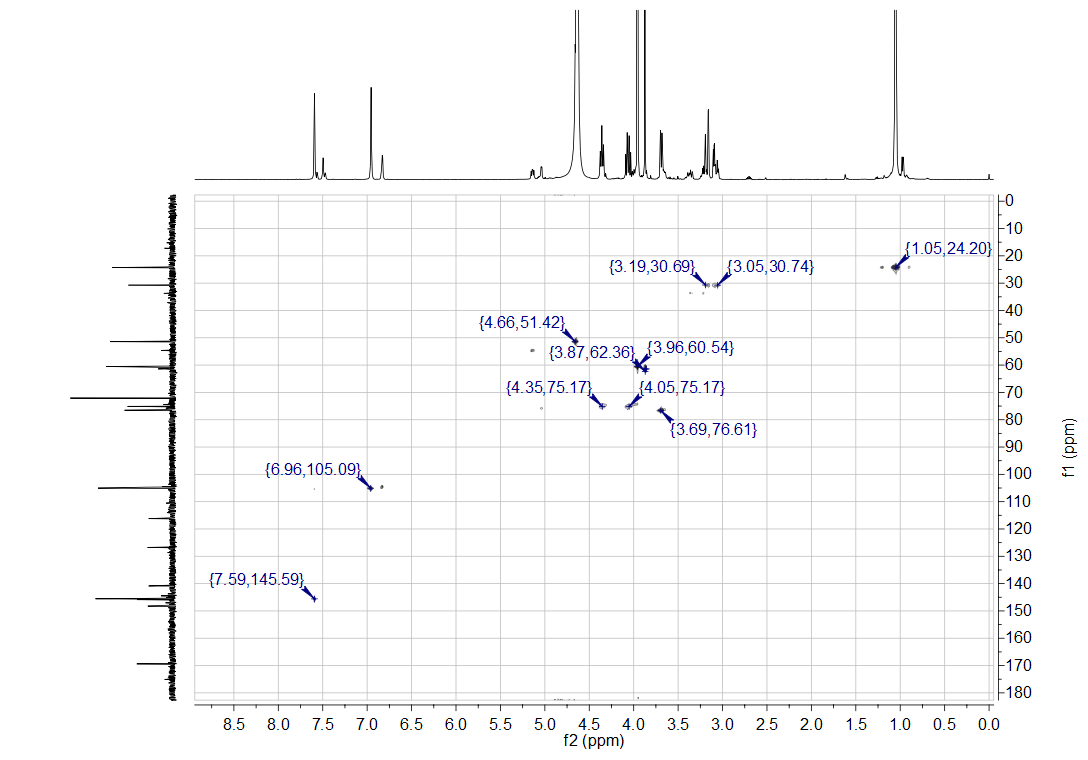
**

**Fig. A16** HSQC spectra of compound **1**

**
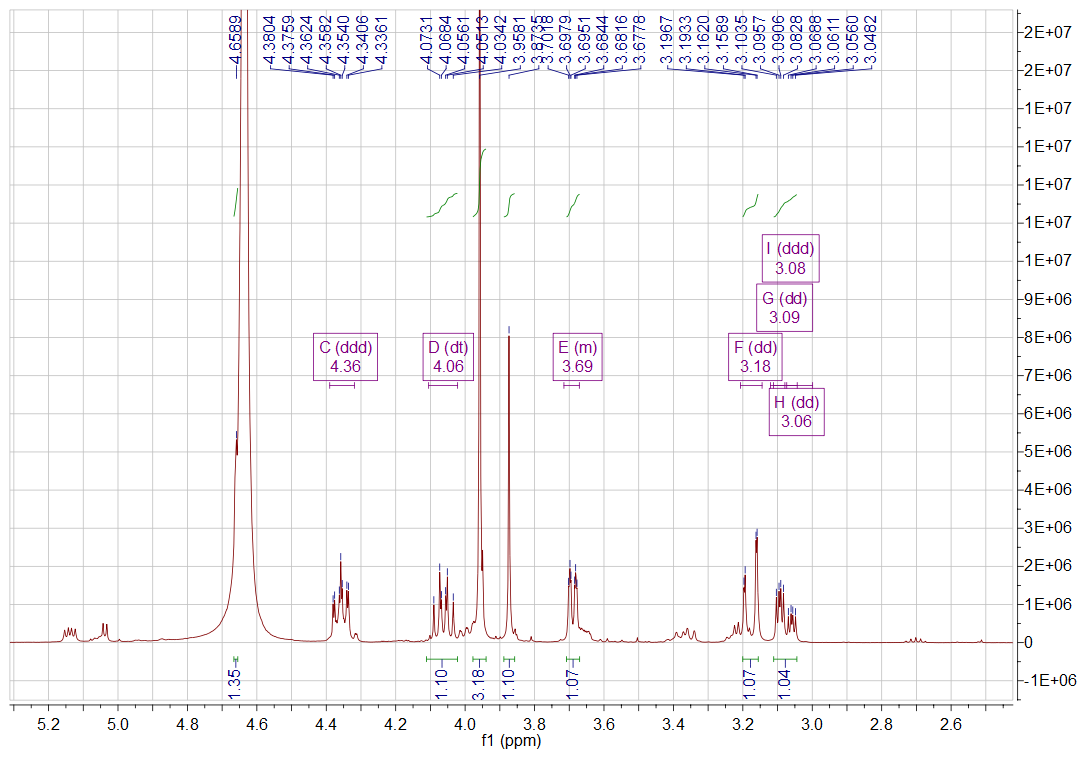
**

**Fig. A17** ^1^H NMR (500 MHz, D_2_O) spectra of compound **1**


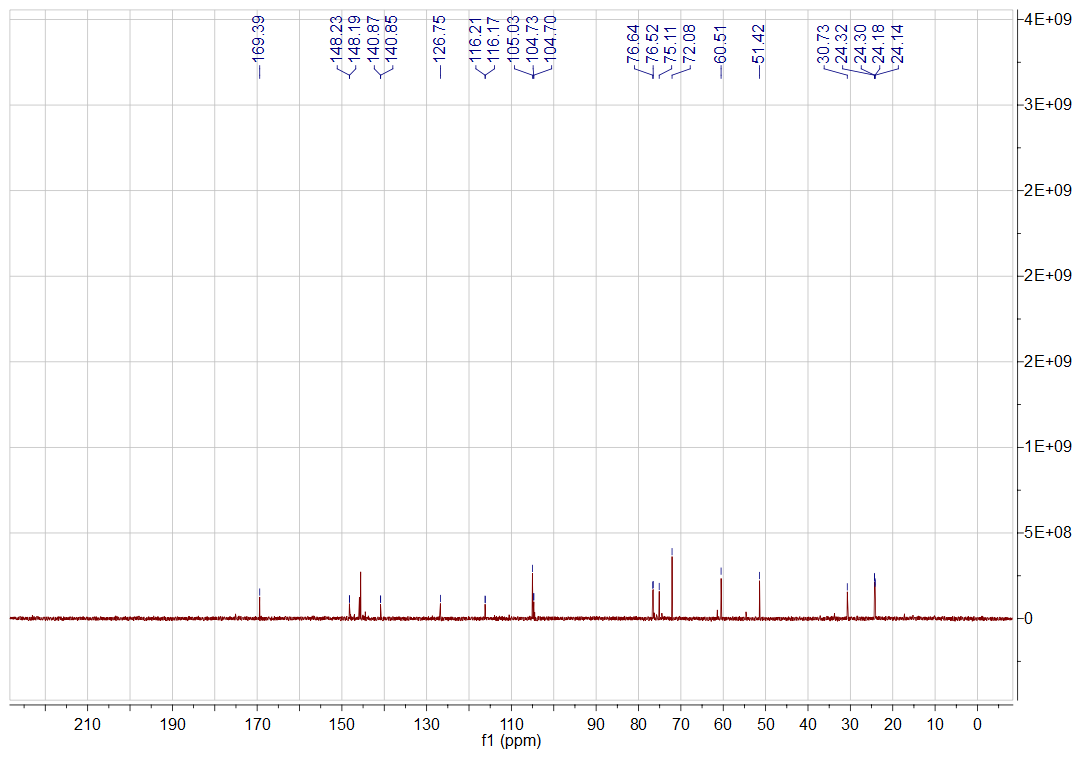


**Fig. A18** ^13^C NMR (125 MHz, D_2_O) spectra of compound **1**

**Table A1.** NMR Spectroscopic Data of compound **3**

| Position | δ_H_ (*J* in Hz) | δ_C_ | HMBC |
| --- | --- | --- | --- |
| 2 | - | 170 | - |
| 3 | 3.34（d, 17.4）  3.23 (d, 17.4), | 31 | 2, 4, 10 |
| 4 | 4.92 (s) | 51 | 2, 3, 5, 9,10 |
| 5 | - | 149 | - |
| 6 | - | 115 | - |
| 7 | - | 156 | - |
| 8 | 7.10(s) | 95 | 6, 7, 9, 10 |
| 9 | - | 149 | - |
| 10 | - | 104 | - |
| 11 | 4.69 (d, 10.2), 4.45 (t, 8.6) | 73 | 5, 12, 13 |
| 12 | 3.84 (m) | 76 | 13, 14, 15 |
| 13 | - | 72 | - |
| 14 | 1.28 (d) | 24 | 12, 13, 15 |
| 15 | 1.28 (d) | 25 | 12, 13, 14 |
| 2’ | 7.75 (s) | 145 | 6, 7, 3’ |
| 3’ | 7.11(s) | 105 | 2’, 5, 6, 7 |

Table A2. B3LYP/6-31g(d) optimized low-energy conformers for (4R,12S)-3

| (4*R*,12*S*)-**3-C1** | (4*R*,12*S*)-**3-C2** | (4*R*,12*S*)-**3-C3** |
| --- | --- | --- |
| 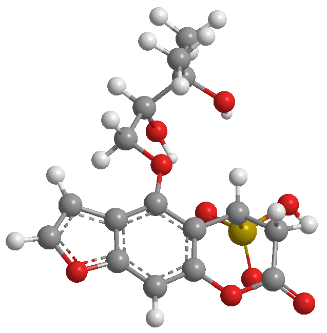  (52.48%) | 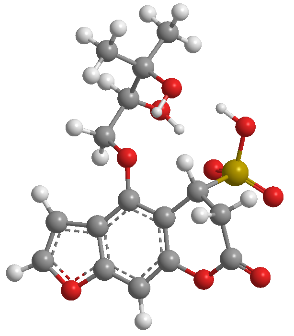  (31.33%) | 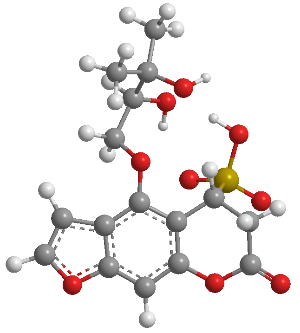  (10.22%) |
| (4*R*,12*S*)-**3-C4** | (4*R*,12*S*)-**3-C5** | (4*R*,12*S*)-**3-C6** |
| 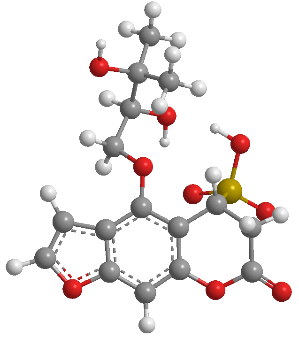  (2.31%) | 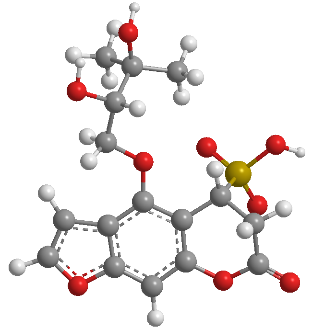  (1.96%) | 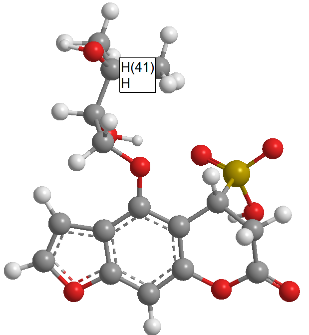  (1.08%) |

Table A3. B3LYP/6-31g(d) optimized low-energy conformers for (4*S*,12*S*)-3

| (4*S*,12*S*)-**3-C1** | (4*S*,12*S*)-**3-C2** | (4*S*,12*S*)-**3-C3** |
| --- | --- | --- |
| 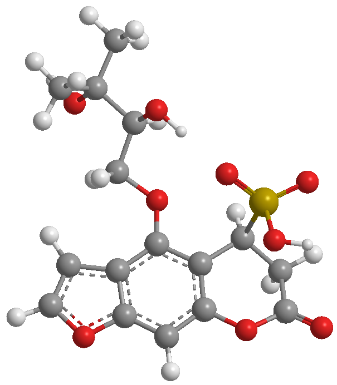  (81.17%) | 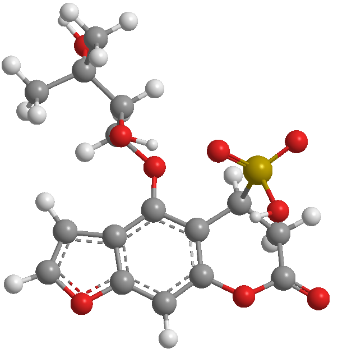  (5.97%) | 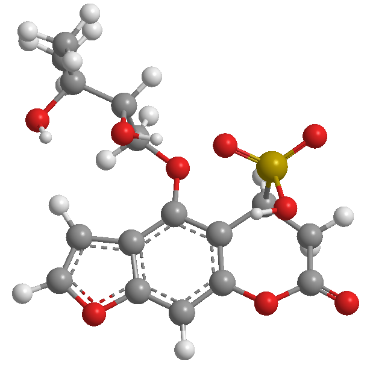  (4.92%) |
| (4*S*,12*S*)-**3-C4** | (4*S*,12*S*)-**3-C5** |  |
| 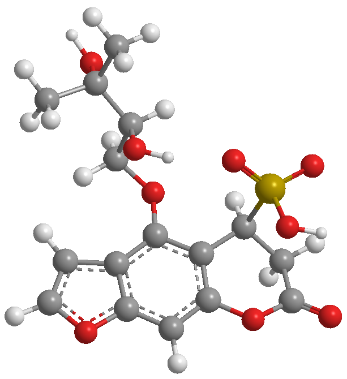  (3.98%) | 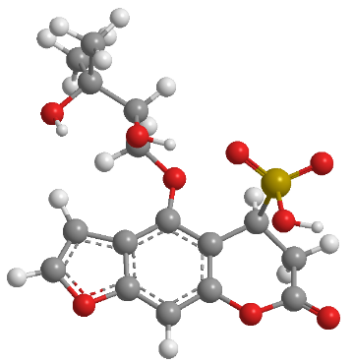  (2.51%) |  |

**Table A4.** *t*-test of cytotoxicity evaluation (*p*, n=3)

| Drug | Dose/µg*mL^-1^ | OXH-S *VS* OXH-N | Extraction-N *VS* Extraction-S |
| --- | --- | --- | --- |
| L02 | 50 | 0.069 | 0.132 |
|  | 100 | 0.043 | 0.178 |
|  | 200 | 0.013 | 0.073 |
| PC12 | 200 | 0.004 | 0.188 |
|  | 400 | 0.046 | 0.908 |
|  | 800 | 0.005 | 0.941 |
| HK2 | 200 | 0.001 | 0.063 |
|  | 400 | 0.025 | 0.229 |
|  | 800 | 0.033 | 0.631 |

**Table A5**. t-test of inflammatory regressions (*p*, n=10)

| Dose/µg*mL^-1^ | OXH-N VS OXH-S | Extraction-N VS Extraction-S |
| --- | --- | --- |
| 55.7 | 0.723 | 0.181 |
| 167 | 0.688 | 0.717 |
| 500 | 0.030 | 0.144 |
